# A selectivity filter in Tim17 mediates intramitochondrial protein sorting

**DOI:** 10.1038/s41467-026-77990-3

**Published:** 2026-10-07

**Authors:** Annika Egeler, Sanjoy Paul, Rosario Valenti, Annika Nutz, Tamara Flohr, Erik Marcel Heller, Markus Räschle, Maya Schuldiner, Gerhard Hummer, Johannes M. Herrmann

**Affiliations:** 1 Cell Biology, University of Kaiserslautern, RPTU, Kaiserslautern, Germany; 2 Department of Theoretical Biophysics, Max Planck Institute of Biophysics, Frankfurt, Germany; 3 Department of Molecular Genetics, Weizmann Institute of Science, Rehovot, Israel; 4 Molecular Genetics, University of Kaiserslautern, RPTU, Kaiserslautern, Germany

**Keywords:** Energy metabolism, Protein translocation, Mitochondria

## Abstract

Mitochondria import hundreds of proteins into the matrix and the inner membrane (IM). The translocase of the IM of mitochondria (TIM23 complex) sorts these proteins using topogenic signals in their structure and directs them into the matrix or inserts them into the IM. How these signals are deciphered is unknown. Here we show that Tim17, the protein-conducting subunit of the TIM23 complex, contains a highly conserved selectivity filter for incoming proteins. Using a fluorescence-based sorting reporter, we identified the mitochondrial oxidoreductase Dmo2 (akin to DMAC1 in humans) as factor for intramitochondrial protein sorting. Molecular dynamics simulations show that a Dmo2-dependent disulfide bond into Tim17 constitutes a negatively charged selectivity filter at the vestibule of the TIM23 complex that arrests membrane proteins and facilitates the lateral insertion. Our work provides a molecular rationale as to how the TIM23 translocase differentiates between soluble and membrane proteins.

## Abstract (web summary)

Tim17 forms the protein-conducting channel of the mitochondrial inner membrane translocase. Here, authors show that the N-terminus of Tim17 constitutes a negatively charged selectivity filter to facilitate the intramitochondrial protein sorting.

## Introduction

**Fig. 1:**
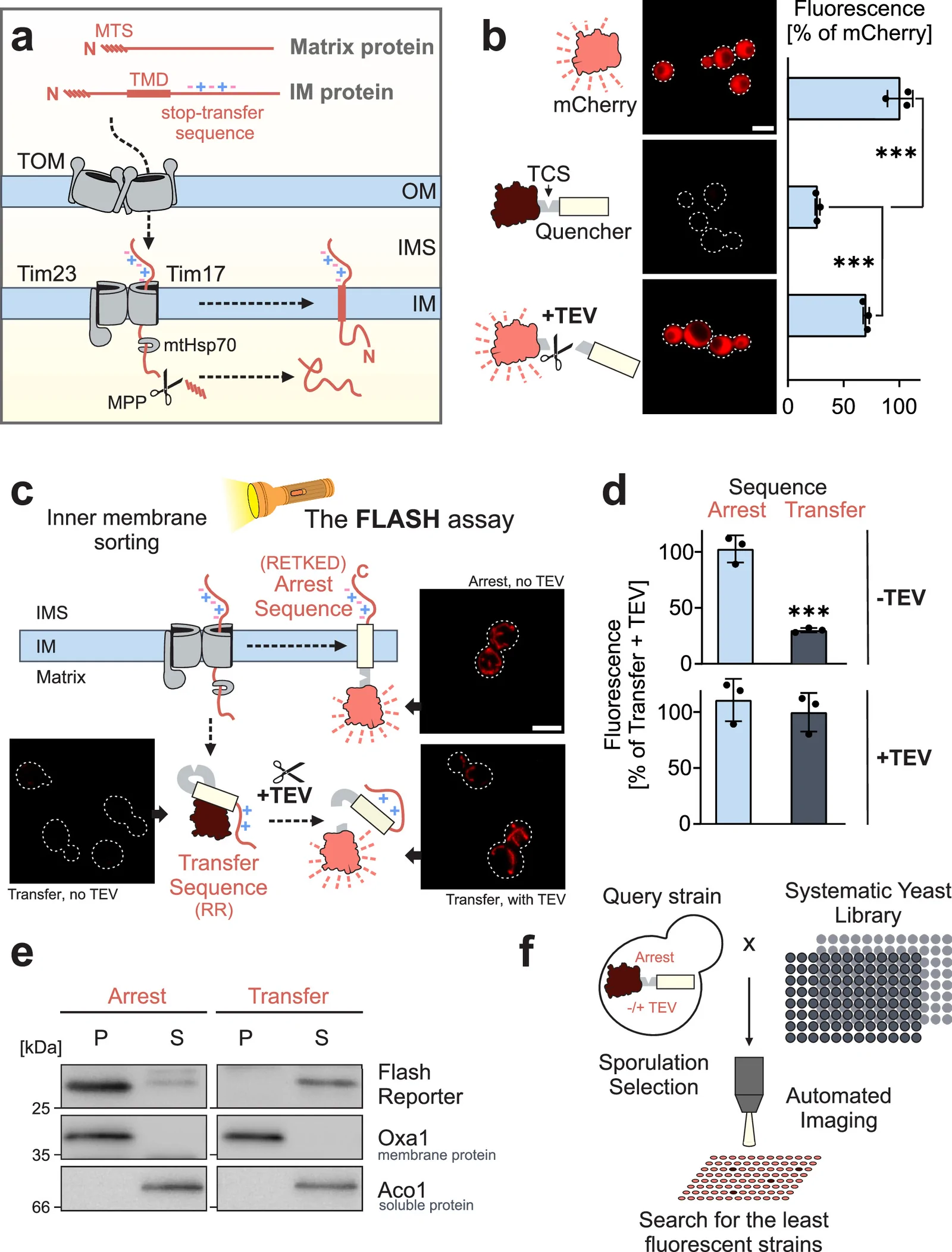
Protein insertion into the IM can be measured by fluorescence dequenching. **a** Schematic representation of the intramitochondrial protein sorting. MPP, mitochondrial processing peptidase; MTS, mitochondrial targeting signal; TMD, transmembrane domain; OM, outer membrane; IM, inner membrane. **b** mCherry as well as a fusion protein of mCherry, TEV cleavage site (TCS) and the quencher domain were expressed in the cytosol of yeast cells using a constitutive *TPI* promoter. For the sample shown in the bottom, the TEV protease was coexpressed with the fusion protein. Fluorescence levels were assessed by microscopy and, in addition, quantified by fluorescence spectrometry. Shown are mean values and standard deviations from three independent measurements. Statistical differences were calculated using a one-way ANOVA. ***Adjusted *P* < 0.001. Scale bar = 5 µm. **c**, **d** Rationale of the FLASH reporter screen. Fusion proteins consisting of a Su9 presequence, mCherry and the quencher domain followed by an RETKED arrest or an RR transfer sequence (Supplementary Fig. 2c) were expressed in yeast cells in the absence or presence of mitochondria-targeted TEV protease. Fluorescence intensities were analyzed by microscopy or spectrometry. Shown are mean values and standard deviations from three independent measurements. Statistical significance was determined using a one-way ANOVA. ***Adjusted *P* = 0.0007. Scale bar = 5 µm. **e** Mitochondria were isolated from cells expressing the quencher fusion proteins. Integral membrane (P, pellet) and soluble (S) fractions were separated by carbonate extraction. The quencher fusions were detected with antibodies against GFP. Oxa1 and Aco1 served as controls for membrane-embedded and soluble proteins, respectively. **f** The Arrest fusion protein without or with the TEV protease were crossed into a systematic yeast library in order to identify mutants with impaired arrest efficiency (low fluorescence) but efficient import (regained fluorescence upon TEV expression).

Mitochondria contain about 1000 (yeast) to 1500 (human) different proteins^1,2^. Except for a very small number of mitochondrially encoded proteins, all these polypeptides are synthesized on cytosolic ribosomes and imported into the organelle. The translocase of the outer membrane (TOM) complex serves as the general entry gate for mitochondrial precursor proteins^3,4^. Most proteins destined to the matrix or the IM carry N-terminal matrix-targeting signals (MTS, also referred to as presequences) that allow them to engage with the translocase of the IM (TIM23) complex^3^. Tim17 serves as the crucial central subunit of the TIM23 translocase: its lateral protein-lipid interface allows proteins to slide through the IM or to insert into the lipid bilayer^5–7^. The IM protein Mgr2 is a non-essential component of the TIM23 complex that suppresses the erroneous lateral insertion of translocation intermediates into the lipid bilayer^6–8^. It is not well understood how the translocation of IM proteins is arrested (Fig. 1a) but clusters of negative charges following the transmembrane domain (TMD) were proposed to serve as a critical arresting element in such stop-transfer signals^9–11^. To better understand the molecular mechanisms and factors that contribute to the sorting of translocation intermediates at the level of the IM, we developed a fluorescence-based assay to monitor the mis-targeting of an IM reporter to the matrix. By screening a genome-wide protein depletion library, we identified yeast mutants with an impaired arrest of IM proteins. We zoomed in on one of the hits, the oxidoreductase Dmo2 (alias Dbi1), showing that it is indeed an important factor at the branchpoint to IM versus matrix targeting by introducing a structural disulfide bond into Tim17^12^. Molecular dynamics (MD) simulations of the substrate-bound TIM23 complex suggested a crucial role of the disulfide bond in positioning the negatively charged N-terminus of Tim17 at the IMS surface of the IM translocase. These charges in Tim17 act as a selectivity filter that arrests the translocation of membrane proteins, thereby facilitating their lateral insertion into the lipid bilayer of the IM^6,7^. Our results provide the first insight into how the specific structure of the TIM23 differentially sorts precursor proteins to the respective subcompartments of mitochondria.

## Results

### FLASH—a fluorescence dequencher reports on mitochondrial membrane insertion

The intramitochondrial sorting of proteins was mainly studied in vitro using radiolabeled precursor proteins that were incubated with isolated mitochondria^13^. This assay is extremely powerful for the dissection of the different steps of the import reaction, but not well suited for systematic screens with large numbers of mutants. We therefore developed an in vivo assay relying on fluorescent proteins and quenching sequences^14,15^. To this end, we used a quencher segment that consists of a hydrophobic helix which—in aqueous environment—tetramerizes and prevents the maturation of fluorescent proteins such as GFP or mCherry (Fig. 1b, Supplementary Fig. 1a-e). However, the insertion into the lipid bilayer of a membrane prevents quenching, inspiring us to employ quencher-based constructs as reporters to measure membrane insertion efficiencies. We fused the quencher domain to mCherry using a linker containing a cleavage site for the tobacco etch virus protease (TEV). Expressing this fusion protein wiped out the fluorescent signal unless TEV expression released the quencher domain from the fluorescent proteins. It should be noted that endogenous yeast proteins do not contain the consensus sequence for the TEV protease and, therefore, the expression of the matrix-targeted TEV did not negatively affect the growth of yeast cells (Supplementary Fig. 1f, g).

Next, we used this dequenching module to design a reporter that monitors protein insertion into the mitochondrial IM which we called FLASH (for FLuorescent Arrest Sensor for the High-throughput analysis of insertion) (Fig. 1c). To this end, we added the mitochondrial presequence of subunit 9 of the ATPase *Neurospora crassa* ATPase to the N-terminus of the mCherry quencher fusion protein. To the C-terminus, we either added the arrest sequence of the IM protein Dld1 of baker’s yeast (RETKED, Arrest reporter) or a non-arresting sequence of a matrix-mistargeted Dld1 mutant (RR, Transfer reporter). Dld1 is a well-characterized model protein that was used to identify the topogenic signals that lead to the arrest and lateral insertion of imported proteins in the IM; the presence of the six residues following the TMD (‘RETKED’) proved to be crucial for the membrane insertion of Dld1^9^. Similar negatively charged sequences are generally found in stop-transfer proteins of the IM and represent the arrest sequences that promote membrane insertion from the TIM23 complex (Supplementary Fig. 2a-c).

Insertion of the quencher domain into the IM resulted in a strong fluorescent signal in the Arrest reporter. By contrast, no fluorescence signal was observed for the non-arresting RR fusion, indicating that the quencher wiped-out the fluorescence signal when the protein was fully imported into the mitochondrial matrix (Fig. 1c, d). Proteolytic release of the mCherry domain by expression of matrix-targeted TEV restored fluorescence in both fusion proteins to comparable levels. The differential sorting of the Arrest and Transfer reporters was also confirmed biochemically by carbonate extraction of mitochondria isolated from these strains (Fig. 1e).

Thus, these fusion proteins are efficient reporters to monitor the intramitochondrial sorting of a membrane protein: the arrest of the Arrest FLASH reporter at the IM led to a strong fluorescent signal. However, its mis-targeting to the matrix should result in fluorescent quenching, so that the FLASH reporter seemed well suited for a systematic screen for mutants in which the arrest of membrane proteins in the TIM23 complex is compromised (Fig. 1f).

### Systematic screening for mutants compromised in membrane insertion

**Fig. 2:**
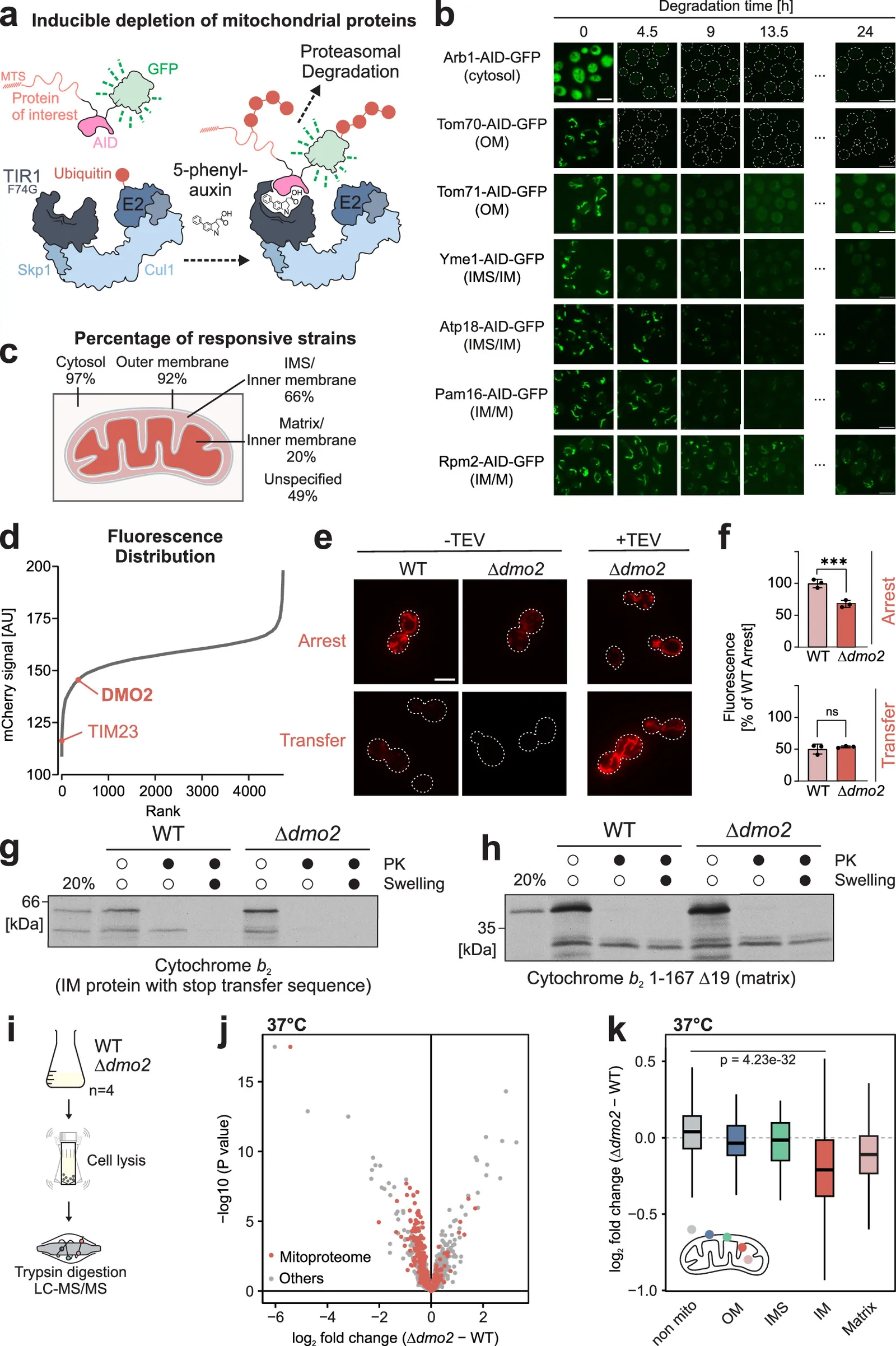
A systematic depletion screen identifies Dmo2 as a critical factor for the arrest of IM proteins. **a** Schematic representation of the induced degradation of fusion proteins. A systematic yeast library expressed all proteins as fusions with an auxin-inducible degron sequence (AID) and the C-terminal GFP for validation. The addition of 5-phenyl-auxin triggered degradation via the TIR1(F74G) ubiquitin ligase protein as previously described^17^. **b** The depletion efficiency in the GFP-tagged protein was controlled by fluorescence microscopy. Shown are examples of proteins of different cellular distribution in the cytosol (control) and the mitochondria (M, matrix) upon different times after addition of 5-phenyl-auxin. Scale bar = 5 µm. **c** The percentage of responsive genes was calculated; displayed is the responsiveness per subcellular compartment during the 24 h treatment with 5-phenyl-auxin. **d** Result of the FLASH reporter screen. The arrest reporter was constitutively expressed in the depletion library. 5-phenyl-auxin was added for 24 h and the GFP and mCherry signals were measured systematically. Shown is the distribution of the mCherry signals and the positions of *TIM23* and *DMO2* are indicated. **e** The mCherry signals in wild type (WT) and *Δdmo2* cells expressing the Arrest and the Transfer reporters were visualized by fluorescence microscopy in the absence and presence of matrix-targeted TEV protease. Scale bar = 5 µm. **f** The FLASH reporter signals in the indicated strains were quantified in a plate-operated fluorescence spectrometer. Shown are mean values and standard deviations of 3 independent replicates. Statistical differences were calculated using a one-way ANOVA. ***Adjusted *P* < 0.001, ns adjusted *P* = 0.867. **g**, **h** Radiolabeled precursor proteins of the IM protein cytochrome *b*_2_ and the matrix-targeted model protein cytochrome *b*_2_Δ19-DHFR were incubated with mitochondria isolated from wild type (WT) or *Δdmo2* cells for 30 min at 37°C. Non-imported proteins were removed by treatment with proteinase K (PK) without or with hypotonic rupturing of the outer membrane (swelling). Mitochondria were reisolated, washed and subjected to SDS-PAGE and autoradiography. The first lane shows 20% of the radiolabeled precursor used per import reaction. **i**, **j** Wild type and *Δdmo2* cells were grown in galactose-based medium at 30 and 37°C, lysed and subjected to mass spectrometry. Four independent biological replicates were analyzed for each condition. The volcano plot shows the protein distribution at 37°C, where the general depletion of mitochondrial proteins is evident. The log_2_ fold changes were calculated using the empirical Bayes moderated two-sided *t*-test implemented in the R limma package^54^, and *P* values were adjusted for multiple testing using the Benjamini–Hochberg method^55^. Data from cultures grown at 30 °C are shown in Fig. Supplementary 4a-c. **k** The log_2_ fold changes of proteins from different intracellular locations were compared between wild type and *Δdmo2* cells grown at 37 °C. Box plots show the distribution of the data. The center line represents the median, while the lower and upper bounds of the box indicate the first and third quartiles, respectively. The whiskers extend to the smallest and largest observations within 1.5 × the interquartile range from the lower and upper quartiles, respectively. Outliers were not displayed. The absence of Dmo2 resulted in a significant reduction of IM proteins, as determined by a two-sided Wilcoxon rank-sum test with continuity correction followed by Benjamini–Hochberg adjustment for multiple testing.

To date most screens for factors relevant for mitochondrial functions were performed by utilizing the yeast deletion collection in which all non-essential genes are knocked out. These collections are often confounded by the emergence of suppressor mutations which are particularly prevalent for mutations affecting mitochondrial function and respiration^16^. To avoid this problem and to be able to test the relevance of essential genes, we made use of a recently developed collection in which yeast genes were systematically fused to an auxin-inducible degron-encoding sequence followed by GFP (AID-GFP domain; Fig. 2a). Proteins containing the AID-GFP extension remain stable unless 5-phenyl-auxin is added, which targets the fusion proteins for very efficient proteasomal degradation via a variant of the auxin-sensitive E3 protein (TIR1^F74G^) that is constitutively expressed in these strains^17^. Cytosolic proteins are efficiently degraded by this method. However, for depletion of mitochondrial proteins which are only accessible to the ubiquitin system during biogenesis, longer degradation periods were found to be necessary (Fig. 2b)^17^. If 5-phenyl-auxin was present for 24 h, many mitochondrial proteins were efficiently depleted, albeit matrix proteins often escaped degradation (Fig. 2c).

We integrated the FLASH reporter into the locus of the non-functional pseudogene *YPRCdelta15* of a yeast cell of mating type MAT a and crossed it into a MAT alpha library of 5110 systematic AID-GFP insertions^17^ using automated mating and sporulation approaches^18,19^. The haploid progeny was then individually screened by microscopy for cells with reduced fluorescence.

In many of these strains, proteins required for protein synthesis were depleted (Supplementary Fig. 3a), explaining the reduced levels of the FLASH reporter. However, the depletion of some mitochondrial proteins resulted in lower fluorescent signals, including Tim23, a central component of the TIM23 complex, and Dmo2, at the time of the screen a non-characterized mitochondrial protein^20,21^(Fig. 2d, Supplementary Fig. 3b, c). TEV expression increased the fluorescence levels in mutants lacking Dmo2 (and the non-characterized protein Emi1) indicating that Dmo2 is indeed important for intramitochondrial protein sorting rather than for mitochondrial protein import per se (Supplementary Fig. 3d).

To confirm the relevance of Dmo2, we applied the FLASH reporter assay in a *DMO2* deletion mutant. Indeed, only low fluorescence signals were detected with the Arrest reporter in this mutant whereas the signal intensity of the Transfer reporter remained unaffected (Fig. 2e, f, Supplementary Fig. 3e). Expression of the matrix-targeted TEV protease restored wild type levels with the Arrest reporter in the mutant, showing that the expression of the sensor is not compromised, but instead its insertion into the IM (Fig. 2e).

In vitro import experiments with isolated mitochondria confirmed the relevance of Dmo2 for the arrest and membrane insertion of IM proteins: Cytochrome *b*_2_ is a well-established IM protein that is imported by a strop-transfer mechanism^22,23^. Upon incubation of the radiolabeled cytochrome *b*_2_ precursor with wild type mitochondria, the precursor reached a protease-inaccessible location. However, selective rupturing of the outer membrane by hypotonic swelling rendered the protein protease-accessible, indicative for its proper arrest in the IM (Fig. 2g). Mitochondria lacking Dmo2 failed to import cytochrome *b*_2_, suggesting a defect in its IM arrest. A mutated version of cytochrome *b*_2_ that lacks the TMD and is therefore directed to the matrix was efficiently imported even when Dmo2 was absent (Fig. 2h).

Finally, we used mass spectrometry (MS) to analyze the effect of Dmo2-loss on the proteome in cells grown at 30 and 37 °C (Fig. 2i, Supplementary Fig. 4a, Supplementary Data 4). Thereby we observed a reduction of many mitochondrial proteins in cells lacking Dmo2, particularly when grown at 37 °C (Fig. 2j, Supplementary Fig. 4b). While proteins of the outer membrane (OM) and IMS showed no significant Dmo2-dependence, many proteins of the IM were profoundly reduced (Fig. 2k, Supplementary Fig. 4c). Thus, our systematic screen identified the protein Dmo2 as a factor that is necessary for the efficient membrane insertion of mitochondrial IM proteins.

### Dmo2 is a conserved IM oxidoreductase

**Fig. 3:**
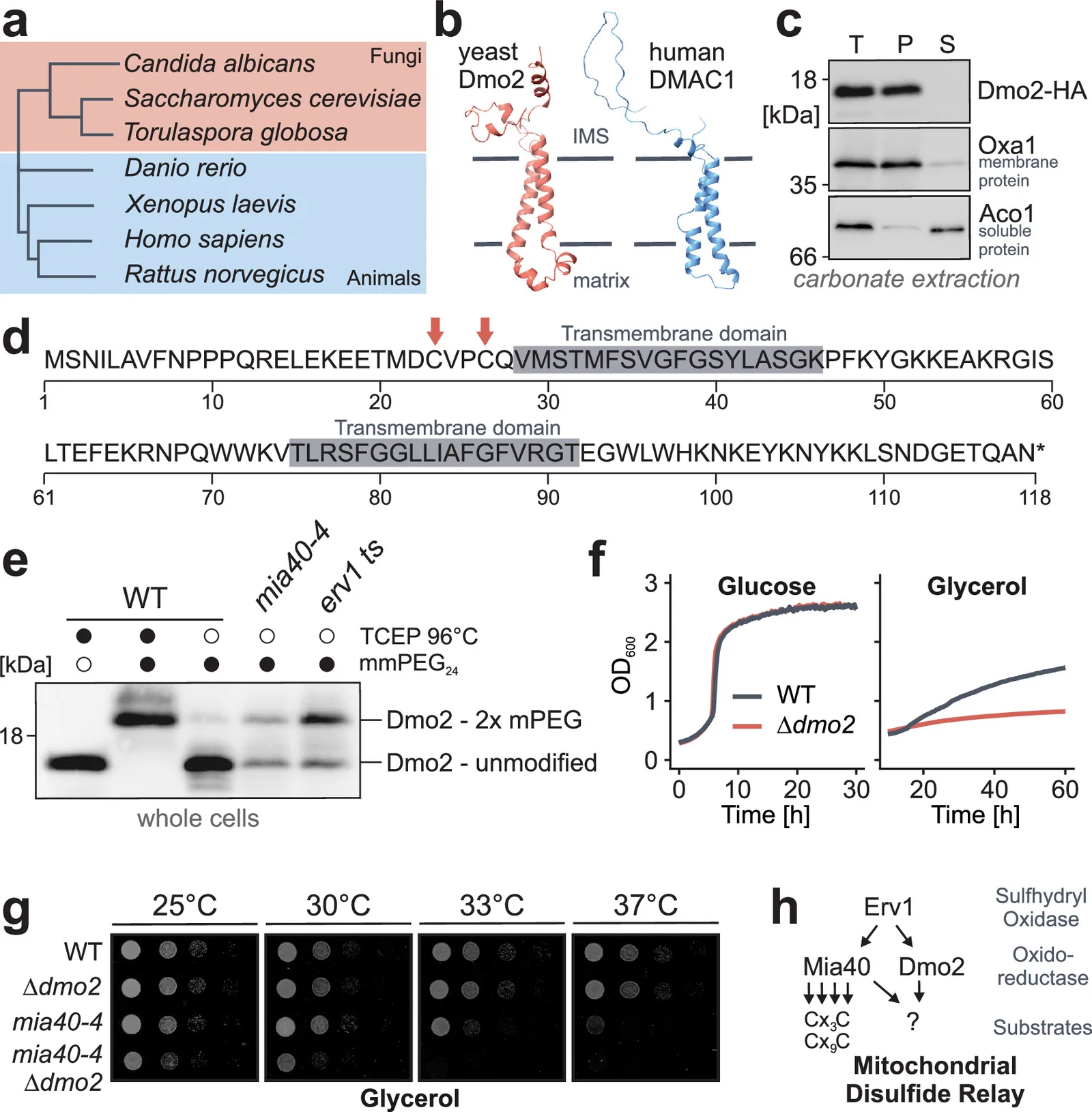
Dmo2 is an evolutionary conserved oxidoreductase of the mitochondrial IM. **a** Sequences of Dmo2/DMAC1 homologs were aligned and used to calculate phylogenetic relationships. See the legend to Supplementary Fig. 5a for details about the different homologs. **b** AlphaFold structures of Dmo1 and its human homolog DMAC1. The predicted position of the IM is indicated. **c** Dmo2 was expressed in yeast cells with a C-terminal hemagglutinin tag. Mitochondria were isolated and either directly applied to SDS-PAGE (T, total) or after carbonate extraction-mediated separation of membrane (P, pellet) and soluble (S) proteins. Dmo2, Oxa1 and Aco1 were detected by Western blotting. **d** The two conserved cysteine residues in the Dmo2 sequence are indicated by arrows. **e** Cell lysates of the indicated strains were treated with 15 mM mmPEG_24_ in the presence or absence of 5 mM TCEP. Proteins were subjected to SDS-PAGE and Western blotting. **f** Wild type and *Δdmo2* cells were grown to log phase and diluted to 0.1 OD before cell growth was monitored in a spectrometer under constant agitation at 37 °C. **g** The indicated strains were grown to log phase before tenfold serial dilutions were spotted onto glycerol-containing plates. **h** Schematic depiction of the mitochondrial disulfide relay.

Dmo2 is a conserved protein that is ubiquitously present in fungi and animals (Fig. 3a, Supplementary Fig. 5a). The human homolog, DMAC1 (also known as TMEM261) was found to be critical for the assembly of complex I^24^ and mitochondrial respiration efficiency; however, the mechanism of its action remained unclear^25^. The AlphaFold structure shows two parallel alpha-helices of hydrophobic nature which are supposed to serve as TMDs (Fig. 3b). Carbonate extraction of isolated mitochondria confirmed that Dmo2 is an integral membrane protein (Fig. 3c). Dmo2 and DMAC1 lack N-terminal presequences but carry internal matrix-targeting signals around their first TMD (Supplementary Fig. 5b). Such signals are typical for proteins which expose their N-termini into the IMS^26,27^. Radiolabeled Dmo2 could be imported into isolated mitochondria, where it reached a protease-inaccessible location, thus supporting its nature as an IM protein (Supplementary Fig. 5c).

The Dmo2/DMAC1 homologs show a universally conserved cysteine-xx-cysteine (CXXC) pair located N-terminally to the first TMD (Fig. 3d, indicated by red arrows, Supplementary Fig. 5a). The CXXC pattern is characteristic for oxidoreductases, i.e. enzymes that catalyze the formation of disulfide bonds^28^. At steady state levels, the two cysteine residues in Dmo2 formed a disulfide bond and were inaccessible to alkylation by the maleimide derivative mmPEG_24_, unless Dmo2 had been pretreated with reductants such as TCEP (Fig. 3e, Supplementary Fig. 4d). Mutants in the mitochondrial sulfhydryl oxidase Erv1 and the oxidoreductase Mia40 showed diminished levels of oxidized Dmo2 as well as reduced Dmo2 levels, indicating that the mitochondrial disulfide relay is critical for Dmo2 biogenesis and/or oxidation.

Deletion of Dmo2 caused a slow-growth phenotype on respiratory carbon sources such as glycerol but not on glucose, symptomatic for mitochondrial dysfunction (Fig. 3f). Moreover, the absence of Dmo2 aggravated the growth defect of temperature-sensitive *mia40-4* cells (Fig. 3g). This negative genetic interaction suggests that Mia40, the oxidoreductase of the IMS, can partially compensate for the loss of Dmo2 (Fig. 3h).

### Dmo2 introduces a disulfide bond into Tim17 that is crucial for the arrest of IM proteins

**Fig. 4:**
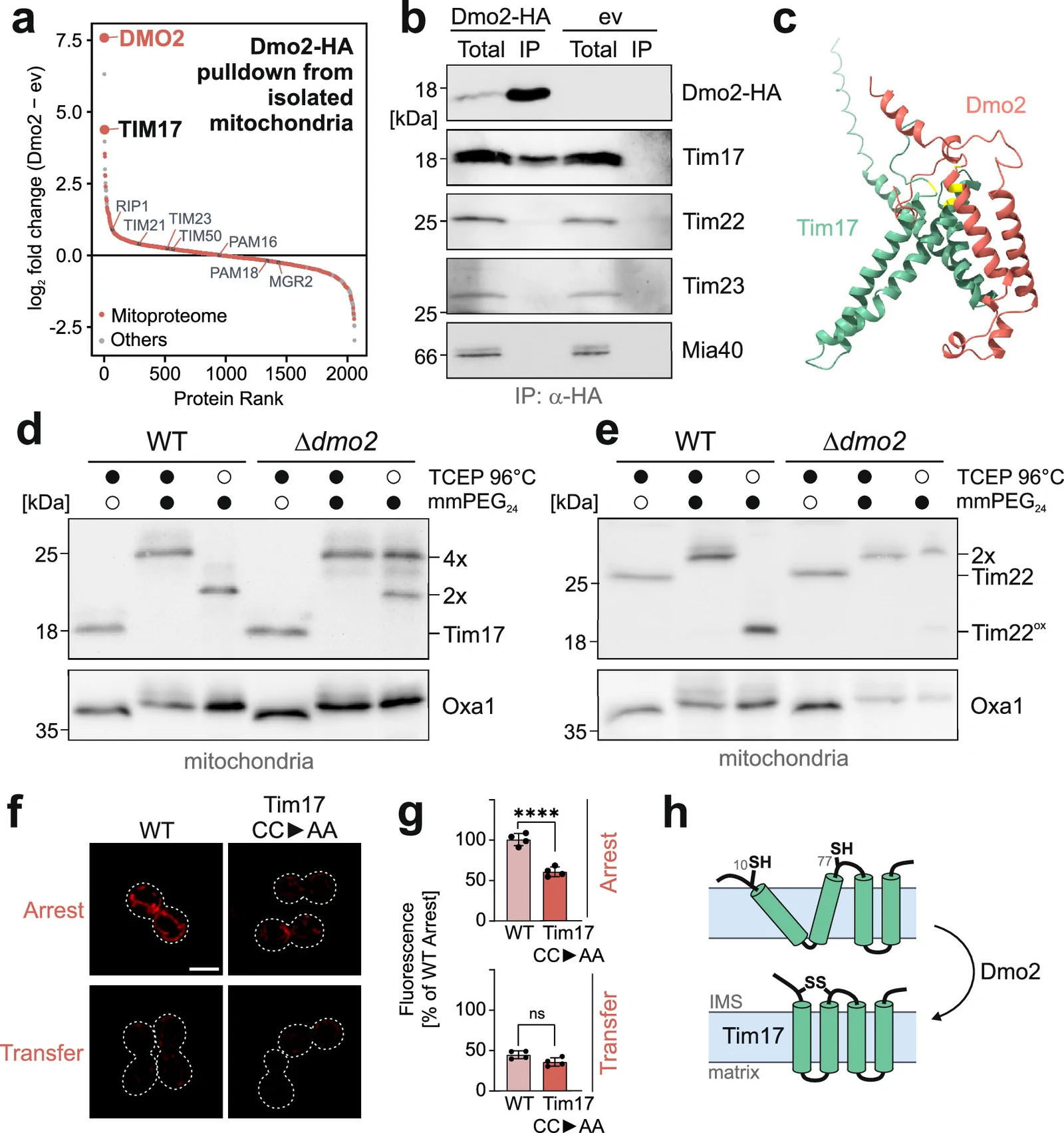
Dmo2 introduces a disulfide bond into Tim17 that is important for IM sorting. **a** Mitochondria (four independent replicates) were isolated from cells containing a Dmo2-HA expressing plasmid or an empty vector for control. Mitochondria were lysed with 0.6% digitonin and incubated with anti-hemagglutinin-coupled magnetic beads. Bound proteins were extensively washed and analyzed by mass spectrometry. **b** The samples described in a were subjected to Western blotting with antibodies against the indicated proteins. **c** AlphaFold model of the Dmo2 Tim17 dimer. Note that the disulfide pair in Dmo2 is juxtaposed to the two disulfide bond-forming cysteine residues in Tim17 (marked in yellow). **d** Mitochondria were isolated from wild type (WT) and *Δdmo2* cells, proteins were precipitated with 12% TCA and treated with 15 mM mmPEG_24_ in the presence or absence of 5 mM TCEP. In *Δdmo2* cells, four cysteine residues can be alkylated in a large fraction of the Tim17 protein, indicative for the absence of its disulfide bond^29^. **e** The redox state of Tim22 was analyzed as described for panel d. Tim22 contains two cysteine residues which are oxidized in wild type cells. In *Δdmo2* cells, the cysteine residues were efficiently alkylated indicating that a large fraction of Tim22 is present in the reduced state. **f**, **g** The FLASH reporters were transformed into wild type and Tim17C10,77 A mutant cells. The cells were grown at 37 °C and the fluorescence intensity was assessed by microscopy and, in addition, quantified by fluorescence spectrometry. Shown are mean values and standard deviations from three independent measurements. Statistical significance was determined using a one-way ANOVA. ****Adjusted *P* < 0.0001, ns adjusted *P* = 0.0578. Scale bar = 5 µm. **h** Schematic depiction of Tim17 oxidation by Dmo2. Reduced Tim17 contains free thiol groups (SH). Oxidation leads to formation of an intramolecular disulfide bond (SS).

In order to identify substrates that are oxidized by Dmo2, we next isolated Dmo2-HA from digitonin-lysed mitochondria and analyzed co-purified proteins by mass spectrometry (Fig. 4a, Supplementary Fig. 6a, Supplementary Data 5). As expected, Dmo2 was highly enriched in the Dmo2-HA eluates. Interestingly, Tim17, the central subunit of the TIM23 complex, was strongly co-isolated with Dmo2. The interaction of Dmo2 with Tim17 was validated by Western blotting of the Dmo2-HA-containing extracts (Fig. 4b). Tim17 contains a highly conserved disulfide bond between cysteines at position 10 and 77^29,30^. AlphaFold models indeed predict a Tim17-Dmo2 complex in which the four cysteine residues are juxtaposed (Fig. 4c), compatible with Dmo2 acting as the Tim17 oxidase. This was then supported by a recent publication reporting Tim17-oxidizing function of Dmo2^12^.

At the time of our screen, the Tim17-oxidizing factor was unknown and we tested whether Dmo2 serves as an oxidoreductase for Tim17. Tim17 contains four cysteine residues: under steady-state conditions, two of these, located in the TMDs, are reduced—while the other two at positions 10 and 77 comprise a disulfide bond. When extracts of isolated mitochondria were treated with mmPEG_24_, only the two reduced cysteines were alkylated unless the extract had been TCEP-treated. In *Δdmo2* mitochondria, a large fraction of Tim17 remained reduced, indicating that Dmo2 facilitates Tim17 oxidation (Fig. 4d). However, it should be noted that even in the absence of Dmo2, a fraction of Tim17 was oxidized, potentially due to some residual oxidation activity of another IMS oxidoreductase, such as Mia40.

Tim23, the assembly partner of Tim17, was not co-isolated with Dmo2. Dmo2 and Tim23 occupy overlapping regions on Tim17 so that the simultaneous binding of Dmo2 and Tim23 to Tim17 seems unlikely (Supplementary Fig. 6b). Thus, Dmo2 presumably introduces the disulfide bond during or subsequent to the insertion of Tim17 into the IM and before Tim17 assembles with Tim23 into the TIM23 complex (Supplementary Fig. 6c). However, it should be noted that the disulfide bond in Tim17 is not absolutely essential for its assembly, and even in the absence of Dmo2 and the disulfide bond in Tim17, Tim17 assembles with Tim23 (Supplementary Fig. 6d).

Interestingly, the Tim22 subunit of the TIM22 translocase, which inserts carrier proteins into the IM, also contains a disulfide bond in a comparable position^30,31^. We therefore performed a mmPEG_24_ shift assay for Tim22 and found that oxidation of Tim22 is also strongly compromised in the absence of Dmo2 (Fig. 4e). In contrast to Tim17, Tim22 was not co-isolated with Dmo2-HA-, suggesting that Dmo2 interacts with Tim22 more transiently.

If the deficient IM arrest of *Δdmo2* mutants is caused by the absence of the disulfide bond in Tim17, one would expect that disulfide-deficient Tim17 mutants would phenocopy *Δdmo2* cells. To test this, we expressed the Arrest and Transfer reporters into wild type and Tim17^CC10,77AA^ cells. The Arrest reporter indeed showed strongly diminished fluorescence levels if Tim17 lacked its disulfide bond (Fig. 4f, g, Supplementary Fig. 6g). It should be noted that Tim17^CC10,77AA^ was present at levels comparable to that of Tim17 (Supplementary Fig. 6e) and still formed a complex with Tim23 (Supplementary Fig. 6f).

Thus, Dmo2 serves as the oxidase for Tim17 (and Tim22) and the Dmo2-mediated formation of the disulfide bond in Tim17 is relevant for the arrest of IM proteins with stop-transfer signals (Fig. 4h). This structural disulfide bond was particularly important at higher temperatures, suggesting that the covalent bond between the cysteines increases the thermostability of Tim17.

### Atomistic MD simulations propose a role for the Tim17 disulfide bond in substrate positioning

**Fig. 5:**
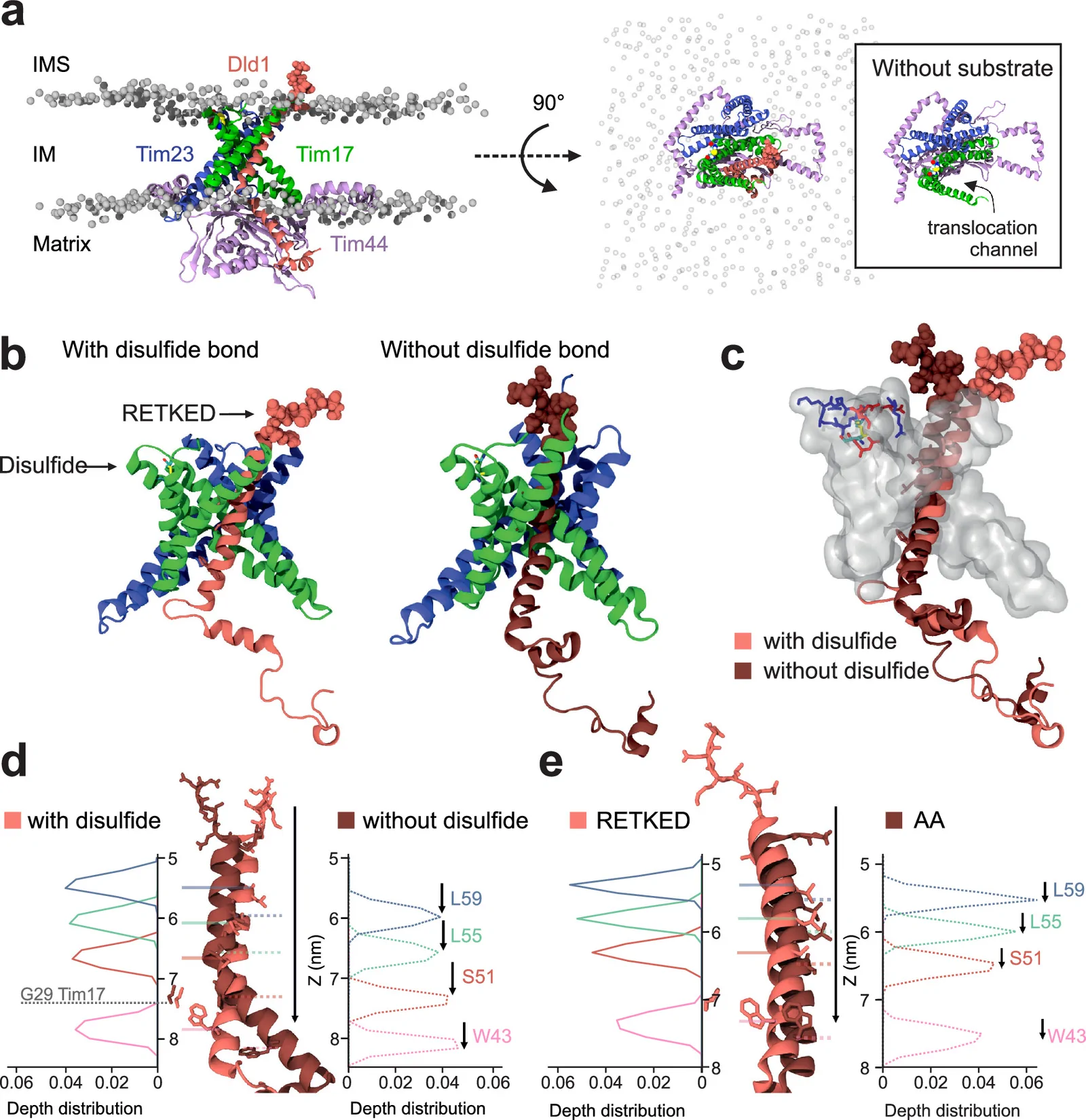
Dynamics and arrest of Dld1 within the Tim17 cavity revealed by atomistic molecular dynamics simulation. **a** Snapshot of the Tim17–Tim23–Tim44 complex embedded in a membrane bilayer (left: side view, right: top view) with Dld1 mimicking the IM after 1 μs of MD simulation (Supplementary Video 1). Tim17 and Tim23 span the membrane, while Tim44 resides in the matrix. Key residues—Cys10 and Cys77 of Tim17, and the RETKED motif of Dld1—are shown as van der Waals spheres. For clarity only phosphate headgroups of the membrane are shown. **b** Cartoon representation of Dld1 within the Tim17 cavity after 1 μs of atomistic simulations of the CG2AT model, shown with (left) and without (right) the Tim17 disulfide bond. The RETKED motif, Cys10 and Cys77 are depicted as sticks. **c** Overlap of the structures shown in b with respect to Tim17. Tim17 is depicted as a transparent surface, while key Dld1 residues (W43, S51, L55, L59) are highlighted as sticks to indicate their vertical positioning in the Tim17 cavity, both with and without the disulfide bond (Supplementary Video 3). The charged (red: acidic, blue: basic) residues of Tim17 (D4, R7, D8, D76, K80, R83, K84, R85, E86, D87) are depicted as sticks. **d** Zoomed in view of Dld1 and the depth distribution profiles of its residues (W43, S51, L55, L59, as in c) in the presence (solid lines) and absence (dotted lines) of the Tim17 disulfide bond, indicating a ∼ 0.4 nm downward (matrix-directed) shift of Dld1 when the disulfide bond is absent. The grey line marks G29 of Tim17, providing a reference for depth assessment. **e** Snapshot of Dld1 and the depth distribution of its residues (similar to d) shows a ∼ 0.2 nm downward shift (Supplementary Video 4) resulting from mutation of the C-terminal charged (RETKED) motif to alanine (AA).

Why is the disulfide bond in Tim17 relevant to IM sorting? To address this, we performed atomistic MD simulations (see Materials and Methods for details) with a substrate-loaded TIM23 complex embedded within a membrane bilayer at ~150 mV membrane potential (Fig. 5a and Supplementary Video 1). The position of the Dld1 substrate matched the preprotein-conducting path that had been recently identified by site-specific cross-linking^5^. In our structure, the TMD of Dld1 resided in the lateral lipid-exposed cavity of Tim17, where it made contacts to residues in the first two TMDs of Tim17 as well as to the surrounding membrane lipids (Fig. 5a and Supplementary Video 1). Our microsecond-scale atomistic simulations reported no significant structural changes of the Tim17 cavity when the disulfide bond between C10 and C77 was reduced (Supplementary Figs. 7a, b, 9a–f and Supplementary Video 2). However, the inter-residue distance between C10 and C77 increased significantly in the absence of the disulfide bond (Supplementary Fig. 7c). In the presence of the disulfide bond, Dld1 maintained a bent structure tightly anchored at the Tim17 cavity (Fig. 5b left). The residues of the Dld1 arrest signal (RETKED) shifted away from the cluster of charged residues (D4, R7, D8, D76, K80, R83, K84, R85, E86, D87) of Tim17 that face the IMS due to electrostatic repulsion. Upon removal of the disulfide bond, the integrity of the charge cluster in Tim17 was disrupted, reducing electrostatic repulsion against the RETKED sequence. Consequently, Dld1 adopted a more linear conformation (Fig. 5b right) compared to its bent structure observed with the intact disulfide bond.

The distinct positioning of Dld1, especially its RETKED motif, relative to the Tim17 cavity became clearer when its orientation in the presence and absence of the disulfide bond was compared (Fig. 5c, Supplementary Figs. 8a-l, 9a-c and Supplementary Video 3). To assess the vertical displacement of Dld1 within the Tim17 cavity, we analyzed the depth distribution (Fig. 5d) of key residues (L59, L55, S51, and W43) along the membrane-spanning helix, using the Tim17 residue G29 as a positional reference. The absence of the Tim17 disulfide bond resulted in a shift of all four Dld1 residues downwards (towards the matrix), while the G29 residue of Tim17 maintained identical positioning in both conditions. These observations demonstrate a prominent matrix-directed downward shift of Dld1 following disulfide bond removal. Quantitative comparison of peak Z-positions revealed an approximately ~0.4 nm displacement of Dld1 towards the matrix in the absence of the disulfide bond compared to the system with intact disulfide bond. In our computational models, the disulfide bond in Tim17 appears to assist in preventing the substrate protein from slipping toward the matrix, providing a plausible model for how lateral transfer into the inner membrane is favored. We further validated this mechanism by replacing the RETKED arrest signal in Dld1 by two alanine residues (AA), while keeping the disulfide bond in Tim17 intact (Supplementary Video 4). Our depth distribution analysis (Fig. 5e) revealed an intermediate (~ 0.2 nm) downward shift of the mutated Dld1 toward the matrix despite an intact disulfide bond, resulting from the RETKED-to-AA mutation, which reduced the electrostatic repulsion from the charge cluster of Tim17.

### The IMS-exposed vestibule of Tim17 forms a charge-based selectivity filter

**Fig. 6:**
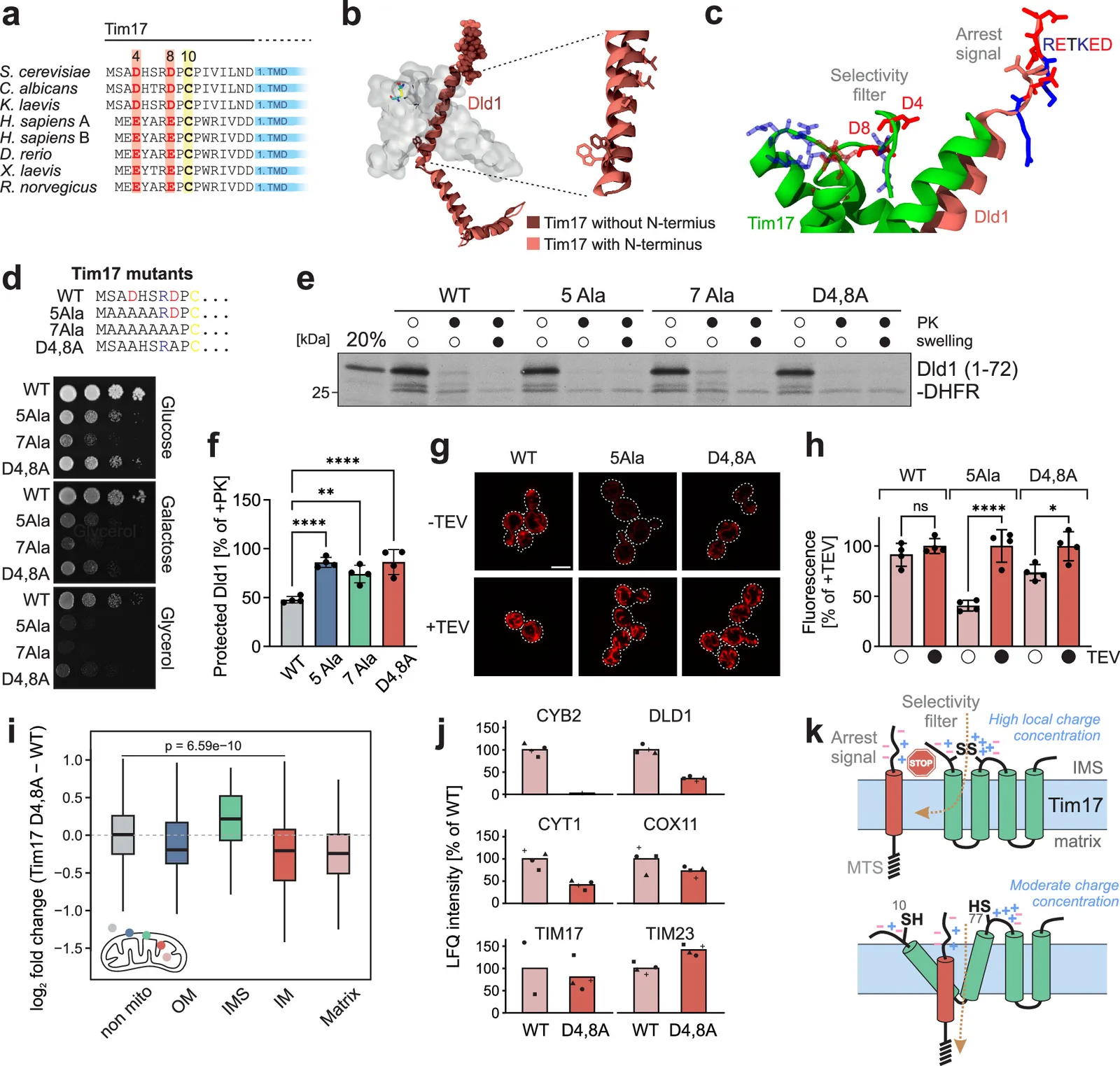
Charged residues in the very N-terminus of Tim17 constitute a selectivity filter for the translocation arrest of IM proteins. **a** Aligned sequences of the IMS-exposed N-terminal segment of Tim17 homologs. The conserved negative charges and the cysteine residue are indicated. **b** Cartoon representation of Dld1 within the Tim17 cavity after 1 μs of atomistic simulations (Supplementary Video 5) of the AT model with and without the N-terminal residues 1–5. The disulfide bond of Tim17 is depicted as sticks, while the RETKED motif of the substrate Dld1 is represented as spheres. Tim17 is shown as a transparent surface, with key Dld1 residues (W43, S51, L55, L59) displayed as sticks (zoomed in view in the right) to compare their vertical positioning in the cavity, including both complete and truncated (residues 1–5 removed) forms. **c** Structural proximity of the very N-terminus of Tim17 (solid sticks) to the RETKED arrest sequence of Dld1, compared to other Tim17 charged residues (transparent sticks). The positions of the aspartates 4 and 8 are indicated as D4 and D8. **d** Mutants of Tim17 with altered N-terminal residues were expressed in a *Δtim17* strain using a plasmid shuffling strategy^32^. Cells were grown to log phase before tenfold serial dilutions were spotted onto plates with the indicated carbon sources and incubated at 37 °C. **e**, **f** Radiolabeled Dld1(1–72)-DHFR was incubated with mitochondria isolated from the indicated strains for 30 min at 37 °C. Mitochondria were treated with proteinase K (PK) under non-swelling or swelling conditions as indicated. In wild type (WT) but not in the mutant mitochondria, a large fraction of the radiolabeled protein became protease-accessible after swelling, indicative for its proper insertion into the IM. The experiment was carried out four times independently and the amount of matrix-targeted protein was quantified. Shown are mean values and standard deviations. Statistical significance was determined using a one-way ANOVA. **** Adjusted *P* < 0.0001, ** adjusted *P* = 0.0022. **g**, **h** Sequences for the Arrest reporter and (where indicated) the TEV protease were integrated into the genome of the indicated tim17 mutants. The cells were grown on glucose medium before the fluorescence was analyzed by microscopy or quantified by fluorescence spectrometry. Shown are mean values and standard deviations from four independent measurements. Statistical differences were calculated using a one-way ANOVA. **** Adjusted *P* < 0.0001, * adjusted *P* = 0.0113, ns adjusted *P* = 0.6309. Scale bar = 5 µm. **i**, **j** The whole cell proteomes of cells expressing the Tim17D4,8 A mutant and of wild type cells were analyzed as described for Fig. 2i-k. The LFQ intensities for selected proteins were compared between wild type (WT) and the Tim17D4,8 A mutant. Values were normalized to WT. **k** Model for the disulfide-stabilized charge cluster on the IMS-exposed vestibule of Tim17. The high charge density in Tim17 is critical for the correct arrest and positioning of preproteins in the TIM23 complex allowing their efficient insertion into the lipid bilayer.

In the published TIM23 structure, the N-terminal 5 amino acid residues of Tim17 had not been resolved. Nevertheless, the N-terminus is exposed to the IMS and is highly conserved (Fig. 6a). Despite its functional importance, its direct role is unknown^32^. Therefore, we included the N-terminal residues in the AT_N1-5_ model (Fig. 6b, Supplementary Fig. 7d). Their presence improved the arrest of the Dld1 substrate by stabilizing the RETKED arrest sequence in the IMS-exposed vestibule of Tim17 (Fig. 6c). In particular, our model proposes a critical role of the conserved negative charge on position 4 of Tim17 (D4) as, together with several charged residues on the Tim17 surface, it prevented the matrix transfer of the arrested substrate (Fig. 6c and Supplementary Video 5). Distance analyses (Supplementary Fig. 8l) revealed that D4 maintained the closest spatial proximity to the RETKED motif during simulations, rationalizing its key contribution in preventing substrate slippage into the matrix.

To prove the relevance of these charged residues, we made point mutants in these residues and replaced the endogenous Tim17 protein by these variants using a plasmid-shuffling approach (Fig. 6d). Mutation of these N-terminal residues strongly compromised cell growth (Fig. 6d, Supplementary Fig. 10a) and mitochondrial respiration rates (Supplementary Fig. 10b) albeit being not critical for its assembly with Tim23 (Supplementary Fig. 10c). Mitochondria isolated from these mutants failed to arrest the Dld1(1–72)-DHFR reporter at the level of the IM and mistargeted it into the matrix, so that it remained protease-inaccessible after hypotonic swelling of the outer membrane (Fig. 6e, f). This impaired protein arrest was confirmed by the Arrest reporter that was mistargeted into the matrix (Fig. 6g, h). We further analyzed the whole cell proteomes of cells expressing these Tim17 mutants or, for control, wild type Tim17 (Fig. 6i, j, Supplementary Fig. 10d, e, Supplementary Data 6). This confirmed that mutation of the conserved negative charges at position 4 and 8 of Tim17 resulted in significant depletion of mitochondrial IM proteins (Fig. 6i), comparable to that observed in the Dmo2 deletion mutant (Fig. 2k). Cytochrome *b*_2_ was among the strongly depleted proteins (Fig. 6j). However, the levels of Tim17 and Tim23 were not depleted and the strong phenotype, therefore, is not caused by a general reduction of the Tim17 levels.

Apparently, the disulfide bond stabilizes the position of the N-terminus of Tim17 with its critical D4 and D8 residues to increase the local charge concentration around the Tim17 vestibule. This high local charge concentration seems critical to properly arrest incoming membrane proteins and facilitate their lateral transfer into the lipid bilayer (Fig. 6k).

## Discussion

The IM of mitochondria has a very high protein content: about one third of all mitochondrial proteins are integral IM proteins which make up about 80% of its mass^1,33^. Most of these proteins reach the IM by lateral insertion from the TIM23 complex, but how the complex distinguishes membrane from matrix proteins has not been well understood. The Tim17 subunit was recently identified as the preprotein-conducting subunit of the TIM23 complex^5,6^. Tim17 forms a lateral cavity that mediates protein translocation along a protein-lipid interface. Negative charges in Tim17 (Supplementary Fig. 7d) within this interface (D17, D76, E126) are critical for the recognition and positioning of the presequence, presumably by aligning with positively charged residues of the amphipathic targeting signal which turn the hydrophobic backsides of presequences towards the lipids^5,34^. For translocation of the mature segments of presequences, Mgr2 (called ROMO1 in humans) can dock onto the lateral opening of Tim17 to improve matrix-targeting^7,8,35^.

In this study, we identified an additional element of the Tim17 protein: charged amino acid residues on the IMS-exposed surface of Tim17 constitute a selectivity filter critical for the recognition and lateral release of integral IM proteins. In total, the N-terminus and first IMS loop of Tim17 contain 9 charged residues. Here, we show that particularly the first two negative charges in positions 4 and 8 (Fig. 6c) are crucial elements in the arrest of preproteins. Our MD simulation data propose that these residues slow down preprotein translocation by interaction and repulsion with the charged arrest signal (Fig. 6k). The negative charges in Tim17 apparently serve as selectivity filter that specifically slows down proteins which have (negatively) charged residues directly following their TMD. It should be noted that the structures of the substrate-bound TIM23 complex shown in this study are models based on the published TIM23 structure and MD simulations; it will be exciting to further characterize the dynamic interaction of different types of preproteins with the import machinery in the future.

The disulfide bond in Tim17 stabilizes the selectivity filter and creates a structural clamp that connects the N-terminus of Tim17 to its IMS loop. This disulfide bond is critical for the position of the arrest signal and the TMD of precursor proteins in the Tim17 cavity, particularly at elevated temperatures. In the absence of the disulfide bond or upon mutation of the negative charges in residues 4 and 8, precursors are not properly arrested and thus become positioned too deep into the open vestibule of Tim17, resulting in their mis-targeting into the mitochondrial matrix. While our MD simulations focused on the model substrate Dld1 and provide a mechanistic framework, they represent simplified working models. Inner membrane proteins broadly harbor clusters of negatively charged residues following their TMD (Supplementary Fig. 2a, b). Still, whether this charge-based arrest mechanism applies universally across diverse membrane precursors remains an important question for future experimental and computational studies.

The extreme N-terminus of Tim17 is highly conserved^32,34,36^ and the charge-based selectivity filter seems to be a universal element of the mitochondrial IM translocase. Interestingly, the Tim22 subunit of the TIM22 translocase, which inserts carrier proteins into the IM, also contains a disulfide bond and several charged residues in a comparable position, suggesting that Tim22 employs the same structural element^30,31^.

The reliable sorting of mitochondrial proteins is of central relevance for mitochondrial function. Defects in mitochondrial protein sorting can give rise to diseases, in particular those that affect muscle and nerve cells^37–40^. Owing to its central role in mitochondrial protein import, Tim17 is crucial for mitochondrial function; interestingly, the overexpression of the human Tim17 isoform TIMM17a was found to suppress defects in cells of patients suffering from mutations of the mitochondrial genome^41^. Dysfunctions in protein import into mitochondria can lead to the depletion of mitochondrial proteins but also to their mistargeting to other cellular locations^42^. The targeting of the mitochondrial inner membrane protein PINK1 is particularly well characterized as it is of central importance in the context of Parkinson’s disease. In healthy cells, PINK1 is imported into the IM, but upon mitochondrial dysfunction, PINK1 accumulates on the outer membrane and serves as a receptor for mitophagy^43,44^. Defects in the sorting of PINK1 can therefore compromise the reliable elimination of non-functional mitochondria^45,46^. It will be interesting to study the relevance of the selectivity filter in Tim17 in the context of diseases in the future.

The oxidoreductase that forms the disulfide bond in Tim17 was unknown. The screen with a novel mutant collection containing auxin-inducible degron-encoding sequences was essential for the identification of Dmo2 as we found that Dmo2 deletion mutants quickly adapt and the initial phenotypes are lost, presumably by rewiring of the mitochondrial disulfide relay system. During the course of this study, Dmo2 was identified as a complex partner with Tim17 in mutants that impaired efficient TIM23 complex assembly^12^. The human Dmo2 homolog DMAC1 was identified in two independent genetic screens^24,25^ and reported to impair assembly of Complex I. Interestingly, the complex I subunit NDUFA11 is structurally related to Tim17 and Tim22 and likewise contains IMS-exposed conserved cysteine residues. Thus, it seems likely that DMAC1 serves as NDUFA11 oxidase, even though this—as well as how Dmo2/DMAC1 are connected to other enzymes of the mitochondrial disulfide relay—still has to be elucidated. It will be exciting to identify the substrate spectrum of the Dmo2 oxidoreductase globally and to study its cooperation with the Mia40-mediated protein oxidation in more depth.

## Methods

### Yeast strains and plasmids

The yeast strains and plasmids used in this study are listed in Supplementary Data 1 and 2, respectively. The *tim17* mutants were generated by plasmid shuffling as described previously^32^. The temperature-sensitive *mia40-4* mutant was provided by A. Chacinska (International Institute of Molecular and Cell Biology, Warsaw, Poland) and the *erv1-ts* mutant was provided by T. Lisowski (University of Düsseldorf, Düsseldorf, Germany).

The pYX142-GFP/mCherry-Q plasmids were generated using Gibson Assembly. GFP and mCherry, lacking the stop codon, were amplified from the plasmids pYX142-mtGFP^47^ and pMitoLOC (Addgene, #58980), respectively. The TEV cleavage site (ENLYFQS) and the quencher peptide (CNDSSDPLVVAASIIGILHLILWILDRL) were introduced via primers used for amplification. Prior to assembly, the pYX142 vector was digested with EcoRI and HindIII.

The pYX142-Su9-mCherry-Q-RETKED (Arrest) and pYX142-Su9-mCherry-Q-RETKED (Transfer) plasmids were generated using the same cloning strategy. The Su9 sequence was amplified from the pYX142-mtGFP plasmid. The quencher together with either the Arrest (…WILD**RETKED**L*) or Transfer (…WIL**RR**L*) sequences were introduced through primers.

The pYX113-TEV and pYX112-Su9-TEV plasmids were generated by classic restriction–ligation cloning. The sequence of the improved TEV protease uTEV3^48^ was amplified from the His6-MBP-uTEV3 plasmid (Addgene, #135464) and inserted into pYX113 using EcoRI and SalI, and into pYX122-mtGFP using BamHI and SalI, thereby replacing GFP while retaining the Su9 presequence.

For genomic integration of the Arrest Quencher and the TEV protease the modular cloning (MoClo) toolkit was used^49^. The Su9-mCherry-Q-RETKED construct (Arrest FLASH reporter), either alone or in combination with Su9-TEV, was fused to a hygromycin resistance cassette and integrated into the *YPRCΔ15* locus. Expression of the fusion proteins was driven by the *TEF1* and *TEF2* promoters, respectively.

To clone pYX142-Dmo2-HA and pGEM4-Dmo2 the *DMO2* sequence was amplified from genomic DNA and inserted into pYX142 and pGEM4 (for in vitro expression) using EcoRI and HindIII. For pYX142, the stop codon was omitted, allowing translation to continue into the HA tag encoded by the vector.

Yeast cells were grown at 30°C either in yeast complete medium (YP) containing 1% (w/v) yeast extract, 2% (w/v) peptone and 2% (w/v) of the respective carbon source or in minimal synthetic respiratory medium containing 0.67% (w/v) yeast nitrogen base and 2% (w/v) of the respective carbon source. To induce the phenotype of the temperature-sensitive mutants, the cells were shifted to 37 °C for 17 h. For induction of *GAL1* 0.5% galactose was added to the medium.

### Systematic genetic array screening

To integrate the Arrest FLASH reporter (with or without the TEV protease) into the AID library^17^, the collection was crossed with the query strains BY4741 Su9-mCherry-RETKED and BY4741 Su9-mCherry-RETKED, Su9-TEV (Supplementary Data 3), by the synthetic genetic array (SGA) method^18,50^. The resulting new collections, validated by check PCR of random clones, were used for performing a high-throughput microscopy screen.

The first screen was performed with the library without protease and with the induction of the AID degron for 24 h. Briefly, a RoToR array pinning robot (Singer Instruments) was used to transfer the strains from agar (minimal synthetic media with glucose (SD) as the carbon source and antibiotic selection), to 384-well plates containing 100 µl of SD with the same selections and 5 µM 5-phenyl-auxin (5-phenyl-1H-indole-3-acetic acid, 5-Ph-IAA, CAS: 168649-23-8; Bio Academia: Product code: 30-003-10) to induced the depletion of the proteins tagged with the AID system. The cultures were grown for 20 h at 30 °C, and 5 µl of each culture was used to seed fresh 384-well plates with 95 µl of SD and 5 µM 5-Ph-IAA, for a final OD_600_ of ∼0.2, using the EVO freedom liquid handler (TECAN). The back-diluted cultures were allowed to grow at 30°C for 4 h, to get the cultures in logarithmic growth phase. Lastly, 50 μl from each well were transferred to a glass-bottom 384-well microscopy plate (Azenta Life Sciences) coated with concanavalin A (Sigma-Aldrich). The cells were allowed to settle and bind the bottom of the plates for 20 min, after which the wells were washed twice with SD with 5 µM 5-Ph-IAA media to remove non-adherent cells. The plates were imaged in the same media, in an automated Olympus inverted fluorescent microscope system. Cells were imaged at room temperature (RT) using an Olympus UPLFLN 60X air lens (NA 0.9), a CSU-W1 Confocal Scanner Unit (Yokogawa), equipped with a 50 µm pinhole disk, and with an ORCA-flash 4.0 digital camera (Hamamatsu), using the ScanR software (version 3.2). The reporter was imaged with a 561 nm laser illumination for the mCherry channel (excitation 561 nm, emission filter 617/73 nm) with 600 ms of exposure and 50% laser intensity; and the protein depletion was followed with a 488 nm laser illumination for the GFP channel (excitation 488 nm, emission filter 525/50 nm) with 800 ms exposure and 50% laser intensity. Brightfield images were also taken, with 200 ms exposure and 100% halogen bulb.

Acquired images were processed by ScanR Analysis to segment individual cells and measure the mean reporter fluorescence per cell. Images were also evaluated manually, and candidates from either method, with reduced mitochondrial fluorescence were selected as putative hits. The hits were picked from both collections, with and without protease, and reanalyzed following the same procedure, but this time with or without the addition of 5-Ph-IAA. Candidates with low fluorescence in the presence of 5-Ph-IAA and the absence of the protease, but with higher fluorescence in every other case were prioritized for follow-ups.

### Growth assays

For spot analysis, the respective yeast strains were grown in liquid media. Yeast cells equivalent to 1 OD_600_ were harvested at the exponential phase. The cells were washed in sterile water and 3 µl of ten-fold serial dilutions were spotted on the respective media followed by incubation at the indicated temperatures. Pictures were taken after different days of the incubation.

Growth curves were performed in a 96 well plate, using the automated SPECTROstar^Nano^ (BMG LabTech). The growth curves started at 0.1 OD_600_ and the OD_600_ was measured every 10 min for 72 h at 37 °C. The mean value of technical triplicates was calculated and plotted in R.

### Oxygen consumption measurements

Oxygen consumption of whole cells was measured using a FirePlate-O2 oxygen meter (PyroScience). Cells were grown in YPGal medium to mid-log phase. Cells corresponding to 1 OD_600_ were harvested by centrifugation (17,000 × *g*, 1 min) and resuspended in 125 µl YPEthanol (2%). 10 µl were dispensed in triplicates into an oxygen sensor microplate, followed by the addition of 190 µl YPEthanol using a RAININ pipetting robot (SelectScience). Oxygen levels were measured immediately after placing the microplate, covered with a dark lid, into the FirePlate-O2 reader. Measurements were performed at 30 °C for 20 min.

### Cell lysates

For whole cell lysates, yeast strains were cultivated in liquid media to mid-log phase. 4 OD_600_ were harvested by centrifugation (17,000 × *g*, 1 min) and resuspended in 200 µl reducing loading buffer (50 mM Tris-HCl pH 6.8, 10% glycerin, 2% SDS, 0.01% bromophenol blue, 100 mM DTT). Cells were transferred to screw-cap tubes containing 1 mm glass beads. Samples were boiled at 96°C for 5 min. Cell lysis was performed using a FastPrep-24 5 G homogenizer (MP Biomedicals, Heidelberg, Germany) with 3 cycles of 20 s, speed 6.0 m/s, 120 s breaks, glass beads. Lysates were stored at -20°C until further use. Equal amounts were resolved via SDS-PAGE.

### Antibodies

The antibodies against Aco1, Actin, Cox2, GFP, Mia40, Oxa1, Tim17, Tim23, Tom70 were raised in rabbits using recombinant purified proteins. The antibodies against Om14 were kindly provided by Thomas Becker (University of Bonn, Germany). The antibodies against Tim22 and Sdh3 were gifts from Nils Wiedemann (University of Freiburg, Germany). The horseradish-peroxidase coupled HA antibody was ordered from Roche (Anti-HA-Peroxidase, High Affinity 3F10, #12 013 819 001). The secondary antibody was ordered from BioRad (Goat Anti-Rabbit IgG (H + L)-HRP Conjugate #172-1019). Antibodies were diluted in 5% (w/v) nonfat dry milk-TBS (Roth T145.2) with the following dilutions: anti-Aco1 1:1000, anti-Actin 1:500, anti-Cox2 1:500, anti-GFP 1:500, anti-Mia40 1:5000, anti-Oxa1 1:1000, anti-Tim17 1:500, anti-Tim23 1:500, anti-Tom70 1:500, anti-Om14 1:500, anti-Sdh3 1:250, anti-HA 1:500, anti-Rabbit 1:10,000.

### Isolation of mitochondria

For the isolation of mitochondria, cells were grown in rich or selective galactose media to mid-log phase and harvested via centrifugation (2,000 × g, 5 min, RT). After a washing step, cells were resuspended in 2 ml per g wet weight MP1 buffer (10 mM Tris pH unadjusted) and incubated for 10 min at 30°C. After washing with 1.2 M sorbitol, yeast cells were resuspended in 6.7 ml per g wet weight MP2 buffer (20 mM KPi buffer pH 7.4, 1.2 M sorbitol, 3 mg per g wet weight zymolyase 20 T (Roth, # 9324.3) and incubated for 1 h at 30°C. Spheroplasts were collected via centrifugation at 4°C and resuspended in ice-cold homogenization buffer (13.4 ml/g wet weight) (10 mM Tris pH 7.4, 1 mM EDTA pH 8, 0.2% fatty acids free bovine serum albumin (BSA), 1 mM PMSF, 0.6 M sorbitol). Spheroplasts were disrupted by 12 strokes with a cooled glass potter. Cell debris was removed via centrifugation at 1500 × *g* for 5 min. The supernatant was centrifuged for 12 min at 17,700 × g for 10 min to collect mitochondria. Mitochondria were resuspended in 1 ml of ice-cold SH-buffer (0.6 M sorbitol, 20 mM Hepes pH 7.4) and diluted to a protein concentration of 10 mg/ml.

### Protein import into mitochondria

Radiolabeled precursor proteins were synthesized using the TNT® Quick Coupled Transcription/Translation System (Promega, # L209A) in the presence of [³⁵S]-methionine. 50 µg isolated mitochondria were resuspended in import buffer (500 mM sorbitol, 50 mM HEPES pH 7.4, 80 mM KCl, 10 mM Mg(OAc)₂, 2 mM KH₂PO₄) supplemented with 2 mM ATP and 2 mM NADH. Where indicated, the membrane potential was dissipated by addition VAO (55.6 µg/ml Valinomycin, 440 µg/ml Antimycin A, 850 µg/ml Oligomycin in ethanol). Samples were preincubated for 10 min at 30°C or 37 °C. Import reactions were started by addition of reticulocyte lysate to a final concentration of 1% (v/v) and incubated for the indicated times. Reactions were stopped by 10-fold dilution in ice-cold SH buffer (0.6 M sorbitol, 20 mM HEPES/KOH, pH 7.4; non-swelling conditions) or in 20 mM HEPES, pH 7.4 (hypotonic swelling conditions). Samples were treated with 100 µg/ml proteinase K for 30 min on ice to remove non-imported precursor proteins. Protease activity was inhibited by addition of PMSF to a final concentration of 2 mM. Mitochondria were reisolated by centrifugation (15 min, 30,000 × *g*, 4 °C), washed with SH/KCl buffer (0.6 M sorbitol, 20 mM HEPES/KOH, pH 7.4, 150 mM KCl) containing 2 mM PMSF, and pelleted again under the same conditions. Pellets were resuspended in reducing sample buffer, heated at 96°C for 5 min and analyzed by SDS–PAGE followed by transfer to nitrocellulose membranes and autoradiography.

### Carbonate extraction

100 µg isolated mitochondria were treated with 500 µl Na_2_CO_3_ (100 mM, pH 10) to extract integral membrane proteins. The samples were kept on ice for 30 min and mixed multiple times. After centrifugation (20 min, 30,000 × *g*, 4°C), the supernatant was transferred into a new reaction tube. The pellet was resuspended in 25 μl reducing loading buffer. Proteins in the supernatant were precipitated with trichloroacetic acid at a final concentration of 12% (v/v) for 2 h at −80°C. Samples were then centrifuged (20 min, 30,000 × *g*, 4°C), the pellet was washed with 1 ml ice-cold acetone, and centrifuged again (20 min, 30,000 × *g*, 4°C). The pellet was dried for 10 min at 30°C and resuspended in 25 μl reducing loading buffer. Samples were resolved via SDS-PAGE.

### Thiol modification assay

The oxidation state of proteins was assessed based on their accessibility to the alkylating reagent methyl-polyethylene glycol-maleimide (mmPEG_24_). For this purpose, either 100 µg of isolated mitochondria or whole cell lysates equivalent to 2 OD_600_ were used. Pellets were resuspended in 100 µl of modification buffer (2% SDS, 80 mM Tris-HCl (pH 7.0), 0.05% bromocresol blue, and 10% glycerol). Samples were then split into aliquots and, where indicated, treated with 5 mM TCEP for 15 minutes at 96°C to reduce disulfide bonds. Next, either DMSO (as a control) or 15 mM mmPEG_24_ (Thermo Scientific #22713) was added to modify reduced cysteine residues (1 h at RT in the dark). Protein samples were subsequently resolved by SDS-PAGE.

### Co-immunoprecipitation

500 µg isolated mitochondria were resuspended in 500 µl lysis buffer (20 mM Tris-HCl, pH 7.4, 50 mM NaCl, 10% glycerol, 0.2 mM EDTA, 0.6% (w/v) digitonin, 1 mM PMSF), incubated on ice and resuspended multiple times. Lysates were clarified by centrifugation (15 min, 30,000 × *g*, 4°C). 20 µl of the supernatant (4% total) was mixed with an equal volume of 2× reducing sample buffer. The remaining supernatant was incubated with 25 µl of equilibrated magnetic beads (Pierce™ Protein A magnetic beads, #88846, or ChromoTek HA-Trap magnetic agarose). For Tim23 co-immunoprecipitation, 4 µl α-Tim23 serum was added to the Protein A beads prior to incubation. Samples were incubated for 1 h at 4°C with end-over-end rotation. Beads were collected with a magnetic stand and the supernatant was discarded. Beads were washed twice with 500 µl ice-cold washing buffer (20 mM Tris-HCl, pH 7.4, 100 mM NaCl, 0.2 mM EDTA, 0.1% (w/v) digitonin, 1 mM PMSF). Bound proteins were eluted by resuspending the beads in 25 µl reducing sample buffer and boiling for 5 min at 96°C. Samples were separated by SDS-PAGE on 16% gels, transferred onto nitrocellulose membranes and analyzed by immunoblotting.

### Sample preparation and mass spectrometric identification of proteins

For quantitative whole-proteome analysis, yeast cells were grown in quadruplicates (*n* = 4) in YPGal at 30°C or 37 °C. 20 OD₆₀₀ were harvested by centrifugation (5000 × *g*, 5 min), washed with ice-cold water, snap-frozen in liquid nitrogen and stored at −80°C. Cell lysis was performed in 6 M guanidinium chloride (GdmCl), 10 mM TCEP-HCl, 40 mM 2-chloroacetamide (CAA; Sigma-Aldrich, #C0267)), and 100 mM Tris-HCl (pH 8.5) using a FastPrep-24 5 G homogenizer (MP Biomedicals; 3 × 30 s, speed 8.0 m/s, 120 s breaks, glass beads; glass beads). Lysates were boiled for 10 min at 96°C, clarified by centrifugation (5 min, 16,000 × *g*, 4°C), and protein concentrations were determined the Pierce BCA ProteinAssay kit (Thermo Scientific, #23225). Proteins (25 µg per sample) were digested overnight at 37 °C with sequencing-grade modified trypsin (Promega, #V5111) and LysC (Wako #125-05061) (each at 1:50, enzyme:protein). Peptides were acidified to pH <2 with trifluoroacetic acid (TFA) and desalted using stage-tips containing SDB-RPS 3 M disks. StageTips were activated with acetonitrile, equilibrated with 30% methanol/1% TFA and washed with 0.2% TFA. After sample loading, peptides were washed with 0.2% TFA and eluted with 80% acetonitrile/5% ammonia, dried in a speed vac and resuspended in buffer A (0.1% formic acid) supplemented with buffer A* (2% acetonitrile, 0.1% TFA).

For co-immunoprecipitation–mass spectrometry experiments, yeast cells were grown in quadruplicates (n = 4) in selective galactose medium. Mitochondria isolation and HA-affinity purification were performed as described above. After the first wash, beads were transferred to a new tube and washed twice with washing buffer II (20 mM Tris-HCl, pH 7.4, 100 mM NaCl, 0.2 mM EDTA, 0.1% (w/v) digitonin). Proteins were digested on-bead by incubation with elution buffer I (2 M urea, 50 mM Tris/HCl pH 7.5, 1 mM DTT, and 5 ng/µl trypsin (Promega, #V5111)) for 1 h at room temperature. The supernatant was collected, and a second elution/digestion step was performed with elution buffer II (2 M urea, 50 mM Tris/HCl pH 7.5, 5 mM 2-chloroacetamide (Sigma-Aldrich, #C0267), 5 ng/µl trypsin) for 30 min at room temperature. Supernatants were combined and incubated overnight at 37°C in the dark. Peptides were acidified and purified with StageTips containing three layers of Empore™ C18 disks^51^. C18 stage tips were activated with 100 µl methanol, 100 µl 0.1% formic acid/ 80% acetonitrile and twice with 100 µl 0.1% formic acid. The acidified peptides were added onto the stage tips and washed with 100 µl 0.1% formic acid. Peptides were eluted with 40 µl 0.1% formic acid/ 80% acetonitrile, dried in a speed vac and resuspended in buffer A (0.1% formic acid) supplemented with buffer A* (2% acetonitrile, 0.1% TFA).

Peptides were separated on 50 cm columns with an inner diameter of 75 μm packed in-house with C18 beads (ReproSil-Pur 120 C18 AQ 1.9 μm, Dr. Maisch) using an EASY nLC 1200 chromatography system (Thermo Fisher Scientific, Germany). Eluting peptides were directly sprayed into a Q Exactive HF mass spectrometer (Thermo Fisher Scientific, Germany). For peptide elution, a gradient prepared from buffer A (0.1% formic acid) and buffer B (80 acetonitrile, 0.1% formic acid) ranging from 2% to 95% buffer B was applied over 3 hours. MS data was acquired using a top 15 method. MS data were processed using MaxQuant Version 2.6.4.0^52^. All other parameters for data acquisition and processing can be retrieved from the dataset available at PRIDE (see below).

### Analysis of mass spectrometry data

The protein groups identified were processed using the R programming language (R version 4.5.1; R Core Team (2022). R: A language and environment for statistical computing. R Foundation for Statistical Computing, Vienna, Austria. Available online at https://www.R-project.org/.). First, the MaxQuant output data was filtered to remove contaminants, reverse hits, proteins identified by site only as well as proteins that were identified in less than three replicates (*N* = 4). For whole cell proteomics, this resulted in 3715 *(Δdmo2*) and 3362 (Tim17 mutants) robustly identified protein groups respectively, whose label-free quantification (LFQ) intensities were log2-transformed, normalized using variance stable normalization^53^ and batchcleaned using limma^54^. For Dmo2-HA co-immunoprecipitation, this resulted in 2053 protein groups. Lastly, missing values were imputed by sampling *N* = 4 values from a normal distribution (seed = 7129456) and using them whenever there are no valid values in a quadruplicate of a condition. For co-immunoprecipitation, only missing values in the empty vector sample were imputed. Different for each data set, the mean of this normal distribution corresponds to the 1% percentile of LFQ intensities. Its standard deviation is determined as the median of LFQ intensity sample standard deviations calculated within and then averaged over each quadruplicate. Proteins were tested for differential expression using limma for the indicated pairwise comparison of samples and a Benjamini-Hochberg procedure was used to account for multiple testing^55^. All relevant test results are listed in Supplementary Data 4-6. Principal component analysis was carried out on the processed LFQ intensities by singular value decomposition. For determination of mitochondrial sublocalization, a merged and manually curated list derived from published data^1,56^ was used.

### Fluorescence microscopy

For microscopy cells were grown to mid-log phase. 1 OD was harvested via centrifugation and cell pellets were resuspended in 30 µl of H_2_O. 3 µl were pipetted onto a glass slide and covered with a cover slip. Manual microscopy was performed using a Leica Dmi8 Thunder Imager. Images were acquired using an HC PL APO100x/1,44 Oil UV objective with Immersion Oil Type A 518 F. For excitation of GFP 475 nm and for mCherry 575 nm were used. Mitochondrial images were taken as Z-stacks. Image analysis was done with the LAS X software and further processing of images was performed in Fiji/ImageJ.

### Fluorescence measurement

Cells were grown to mid-log phase in liquid medium and 4 OD_600_ of a yeast culture were harvested by centrifugation (5000 × *g*, 5 min). The pellet was resuspended in 400 µl sterile water. Next, 100 µl (=1 OD_600_) were transferred to a black flat-bottomed 96-well plate (BD Falcon, Heidelberg, Germany) in technical triplicates. The plate was centrifuged at 30 × *g* for 5 min to sediment the cells and fluorescence (mCherry: excitation 570 nm, emission 620 nm; GFP: excitation 485 nm, emission 530 nm) was measured using a CLARIOStar fluorescence plate reader (BMG-Labtech, Offenburg, Germany). Yeast cells not expressing any fluorescent protein were used for background subtraction. Statistical analyses and bar graph generation were performed using GraphPad Prism version 11.0.0.

### Computational analysis of charge over-representation

To compare whether (negative) charges are over-represented in amino acid residues occurring after TMDs we compared the number of charged residues in protein sequences against the number of charged residues in the entire yeast proteome. We downloaded the FASTA file of the *S. cerevisiae* proteome from UniProt (accession: UP000002311) using the UniprotR R-package^57^. We applied a sliding window over the amino acid residues visualized, using a window size of 3 residues, and counted the number of charged, negatively charged, and uncharged residues. Using the window and whole proteome counts we constructed 2×2 contingency tables, comparing the number of charged against uncharged residues for each window. Additionally, we calculated contingency tables comparing the number of negatively charged residues against uncharged residues in the same manner. To obtain odds ratios and its *p*-values, we applied one-sided Fisher’s Exact Tests on the contingency tables constructed from each window. The odds ratio thus represents the odds of an amino acid residue being (negatively) charged in a given window in relationship to the whole yeast proteome. The p-values of the windows are not statistically independent, as the windows are overlapping. We therefore used the method introduced by Benjamini and Yekutieli for multiple testing correction^58^.

### Modeling, molecular dynamics simulations and visualization

An atomic model of the Dld1–Tim17–Tim17–Tim44 complex was generated using AlphaFold 3^59^. For Dld1, only residues up to the arrest signal (RETKED) were included. Due to the absence of a substrate and missing coordinates for regions such as TIM44 residues 194–254 and the N-terminal residues 1–5 of Tim17 in the cryo-EM structure reported by Sim et al.^6^, we considered the AlphaFold-derived model for our simulations. The TIM23 complex with the Dld1 substrate was embedded in a model yeast IM with a composition of 38% DOPC, 24% DOPE, 4% DOPS, 16% POPI, 16% CDL2, 2% POPA (Supplementary Table 1) and a size of ~15.5 nm x 15.5 nm. Tim17 and Tim23 spanned the membrane, while Tim44 resided at the matrix face. The amphipathic helix of Tim44 (residues 255–265) initially protruded into the membrane but gradually reoriented to lie parallel to the membrane–water interface as the simulation progressed. To enhance sampling and assess the statistical robustness of our findings, we performed two sets of simulations. In one set (CG2AT model), the atomistic structure was first converted to a coarse-grained representation using the Martini 2.2^60^ force field, and simulated for ~2 μs with GROMACS 2021.5^61^. The coarse-grained structure was then converted back to an all-atom representation using the CG2AT2 tool^62^, followed by a 1 μs production MD simulation employing the CHARMM36m^63^ force field. A total of four replicas were seeded from distinct time points along the coarse-grained trajectory and backmapped for atomistic simulations. In another set (AT model), the atomistic model of the protein complex was inserted into an atomistic membrane bilayer (composition and size same as before) using CHARMM-GUI^64,65^. The system was then solvated with TIP3P water, and a physiological salt concentration (150 mM NaCl) was maintained. Energy minimization, equilibration, and production runs were subsequently performed using the solvated and ionized structure (Supplementary Table 2 and Supplementary Fig. 11). Minimization was conducted for 5000 steps using the steepest descent algorithm, followed by equilibration under NVT conditions for 250 ps and subsequently under NPT conditions for approximately 2 ns. During equilibration, harmonic position restraints were initially applied to the heavy atoms of the protein, which were then gradually released. The equilibrated system without restraints was subjected to 4 independent production runs in the NPT ensemble, each for 1 µs and without position restraints. The integration timestep was 2 fs. The system temperature and pressure were maintained at 303 K and 1 bar, respectively using the Nose-Hoover thermostat^66,67^ and a semi-isotropic Parinello-Rahman barostat^68^. Long range electrostatic interactions were treated using the particle-mesh Ewald (PME)^69^ algorithm. The LINCS algorithm was used to constrain covalent bonds involving hydrogen atoms. In all the atomistic simulations, an electric field of 0.01 ${\mbox{V}}\bullet {\mbox{n}}{{\mbox{m}}}^{-1}$ was applied^70^ to generate a ~ 150 ${\mbox{mV}}$ membrane potential across the membrane mimicking the physiological conditions across the IM. Depth distribution profiles for Dld1 residues and Tim17 residue G29 were generated using gmx density (dens option ‘number’), with the analysis restricted to backbone atoms. In the above systems, with and without disulfide-bond, residues 1–5 at the N-terminus of Tim17 were excluded due to their poor resolution in the cryo-EM density and their location within a flexible loop region. However, we also built a model with the N-terminal residue 1–5 of Tim17 included, referred to as AT_N1-5_ model, and compared the movement of Dld1 when its arrest signal (RETKED) is mutated to alanine (termed as AT_N1-5|AA_ model). This also allowed us to investigate how removal of N-terminal residues 1–5, which includes D4, influenced Dld1 dynamics. Simulation trajectories were analyzed with VMD^71^(Supplementary Table 3). Videos were made using the Molywood software package^72^.

### Statistics and reproducibility

All experiments were independently repeated at least three times unless otherwise stated. Independent biological replicates were used throughout the study and representative experiments or images shown in the figures were selected from repeated experiments with consistent outcomes.

### Reporting summary

Further information on research design is available in the Nature Portfolio Reporting Summary linked to this article.

## Supplementary information

Supplementary Information

Description of Additional Supplementary Files

Video 1

Video 2

Video 3

Video 4

Video 5

Dataset 1

Dataset 2

Dataset 3

Dataset 4

Dataset 5

Dataset 6

Dataset 7

Reporting Summary

Transparent Peer Review file

## Source data

Source Data

## Data Availability

The mass spectrometry proteomics data generated in this study (see also Supplementary Data 4-6) have been deposited to the ProteomeXchange Consortium via the PRIDE^73^ partner repository with the following dataset identifiers: PXD068062 (Supplementary Data 4), PXD078122 (Supplementary Data 5), and PXD078078 (Supplementary Data 6). The initial and final coordinate files, topology files, and run configuration files used for the molecular dynamics simulations have been uploaded to Zenodo and are accessible at: https://zenodo.org/records/20133271. All other data are available from the lead contact on request. Source data are provided with this paper.
