# Supplementary material for: A selectivity filter in Tim17 mediates intramitochondrial protein sorting
Source: Nat Commun. 2026 Oct 7;17:11424. doi: 10.1038/s41467-026-77990-3 (PMC13736910; doi:10.1038/s41467-026-77990-3)
Supplement: Supplementary file 1 [file 41467_2026_77990_MOESM15_ESM.pdf]

nature portfolio

Corresponding author(s): Johannes Herrmann

Last updated by author(s): Aug 4, 2026

Reporting Summary

Nature Portfolio wishes to improve the reproducibility of the work that we publish. This form provides structure for consistency and transparency in reporting. For further information on Nature Portfolio policies, see our Editorial Policies and the Editorial Policy Checklist.

Statistics

For all statistical analyses, confirm that the following items are present in the figure legend, table legend, main text, or Methods section.

n/a	Confirmed	
☐	☒	The exact sample size (n) for each experimental group/condition, given as a discrete number and unit of measurement
☐	☒	A statement on whether measurements were taken from distinct samples or whether the same sample was measured repeatedly
☐	☒	The statistical test(s) used AND whether they are one- or two-sided<br>*Only common tests should be described solely by name; describe more complex techniques in the Methods section.*
☐	☒	A description of all covariates tested
☐	☒	A description of any assumptions or corrections, such as tests of normality and adjustment for multiple comparisons
☐	☒	A full description of the statistical parameters including central tendency (e.g. means) or other basic estimates (e.g. regression coefficient) AND variation (e.g. standard deviation) or associated estimates of uncertainty (e.g. confidence intervals)
☐	☒	For null hypothesis testing, the test statistic (e.g. F, t, r) with confidence intervals, effect sizes, degrees of freedom and P value noted<br>*Give P values as exact values whenever suitable.*
☒	☐	For Bayesian analysis, information on the choice of priors and Markov chain Monte Carlo settings
☒	☐	For hierarchical and complex designs, identification of the appropriate level for tests and full reporting of outcomes
☒	☐	Estimates of effect sizes (e.g. Cohen's d, Pearson's r), indicating how they were calculated

Our web collection on statistics for biologists contains articles on many of the points above.

Software and code

Policy information about availability of computer code

Data collection	Fluorescence measurements: CLARIOStar (BMG LabTech control sofware and MARS data analysis software, www.bmglabtech.com)<br>Fluorescence microscopy: Leica Dmi8 Thunder Imager (Leica Application Suite X version 3.7.5, www.leica-microsystems.com) and Olympus IX83 (ScanR version 3.2, www.evidentscientific.com)<br>Oxygen Consumption: FirePlate-O2 optical oxygen meter (PyroScience Workbench version 1.5.7.2519, www.pyroscience.com)<br>Growth curves: SPECTROstarNano (BMG LabTech control software and MARS data analysis software, www.bmglabtech.com)<br>Chemiluminiscent imager: ChemiDoc MP (Bio-Rad ImageLab version 6.1, www.bio-rad.com)<br>MD Simulations: GROMACS (version 2021.5, gromacs.org)
Data analysis	Fluorescence measurements: GraphPad Prism version 11.0.0<br>Fluorescence microscopy: Fiji/ Image J version 1.52i (imagej.net/software/fiji)<br>Mass spectrometry: MaxQuant version 2.6.4.0<br>Mass spectrometry, growth curves and oxygen consumption rates: Custom algorithms were developed with R (version 4.5.1)<br>MD simulations (analysis and visualization): VMD (version 1.9.4a57)

For manuscripts utilizing custom algorithms or software that are central to the research but not yet described in published literature, software must be made available to editors and reviewers. We strongly encourage code deposition in a community repository (e.g. GitHub). See the Nature Portfolio guidelines for submitting code & software for further information.

Data

Policy information about availability of data

All manuscripts must include a data availability statement. This statement should provide the following information, where applicable:

- Accession codes, unique identifiers, or web links for publicly available datasets
- A description of any restrictions on data availability
- For clinical datasets or third party data, please ensure that the statement adheres to our policy

The mass spectrometry proteomics data have been deposited in the ProteomeXchange Consortium via the PRIDE partner repository under the dataset identifiers PXD068062, PXD078122 and PXD078078.
The initial and final coordinate files, topology files, and run configuration files used for the molecular dynamics simulations have been uploaded to Zenodo and are accessible at: https://zenodo.org/records/20133271

Research involving human participants, their data, or biological material

Policy information about studies with human participants or human data. See also policy information about sex, gender (identity/presentation), and sexual orientation and race, ethnicity and racism.

Reporting on sex and gender	not applicable
Reporting on race, ethnicity, or other socially relevant groupings	not applicable
Population characteristics	not applicable
Recruitment	not applicable
Ethics oversight	not applicable

Note that full information on the approval of the study protocol must also be provided in the manuscript.

Field-specific reporting

Please select the one below that is the best fit for your research. If you are not sure, read the appropriate sections before making your selection.

[x] Life sciences [ ] Behavioural & social sciences [ ] Ecological, evolutionary & environmental sciences

For a reference copy of the document with all sections, see nature.com/documents/nr-reporting-summary-flat.pdf

Life sciences study design

All studies must disclose on these points even when the disclosure is negative.

Sample size	Sample sizes were not determined using formal statistical methods, but were based on prior experience with this specific type of experiment and on commonly used sample sizes reported in comparable studies within the field. All key experiments were performed independently at least three times, allowing assessment of variability between replicates and ensuring consistency with previously published work.
Data exclusions	No data were excluded from the analyses.
Replication	Experiments were performed in at least 3 independent biological replicates unless indicated differently. For most experiments, mean and standard deviation or the individual results of all replicates are shown. For western blot experiments, representative results are shown. Replication was successful in all attempts.
Randomization	The clones used for individual experiments were picked randomly from a cultural streak for single colony.
Blinding	Not relevant for this study, as it does not include animals and/or human research participants.

Reporting for specific materials, systems and methods

We require information from authors about some types of materials, experimental systems and methods used in many studies. Here, indicate whether each material, system or method listed is relevant to your study. If you are not sure if a list item applies to your research, read the appropriate section before selecting a response.

Materials & experimental systems

n/a	Involved in the study	
☐	☒	Antibodies
☐	☒	Eukaryotic cell lines
☒	☐	Palaeontology and archaeology
☒	☐	Animals and other organisms
☒	☐	Clinical data
☒	☐	Dual use research of concern
☒	☐	Plants

Methods

n/a	Involved in the study	
☒	☐	ChIP-seq
☒	☐	Flow cytometry
☒	☐	MRI-based neuroimaging

Antibodies

Antibodies used	The antibodies against Aco1, Actin, Cox2, GFP, Mia40, Oxa1, Tim17, Tim23, Tom70 were raised in rabbits using recombinant purified proteins. The antibodies against Om14 were kindly provided by Thomas Becker (University of Bonn, Germany). The antibodies against Tim22 and Sdh3 were gifts from Nils Wiedemann (University of Freiburg, Germany). The horseradish-peroxidase coupled HA antibody was ordered from Roche (Anti-HA-Peroxidase, High Affinity 3F10, #12 013 819 001). The secondary antibody was ordered from BioRad (Goat Anti-Rabbit IgG (H+L)-HRP Conjugate #172-1019). Antibodies were diluted in 5% (w/v) nonfat dry milk-TBS (Roth T145.2) with the following dilutions: anti-Aco1 1:1000, anti-Actin 1:500, anti-Cox2 1:500, anti-GFP 1:500, anti-Mia40 1:5000, anti-Oxa1 1:1000, anti-Tim17 1:500, anti-Tim23 1:500, anti-Tom70 1:500, anti-Om14 1:500, anti-Sdh3 1:250, anti-HA 1:500, anti-Rabbit 1:10,000.
Validation	All antibodies raised on yeast proteins were tested with respective yeast mutants. In the case of non-essential genes, deletion mutants were used. In case of essential genes, either over-expression strains were used or mutants in which the respective proteins were larger or smaller due to deleted regions or fusions. Thereby the specificity of all antibodies against yeast proteins could be confirmed by Western blotting. Secondary antibodies were obtained from commercial sources and had been verified by Biorad.

Eukaryotic cell lines

Policy information about cell lines and Sex and Gender in Research

Cell line source(s)	No mammalian or established eukaryotic cell lines were used in this study. Instead, experiments were conducted using Saccharomyces cerevisiae strains, which are listed with their genotypes and sources in Table S1.
Authentication	Mutant strains were confirmed by control PCR to confirm the correct genetic locus of the insertion or deletion. Expression levels were tested by Western Blotting.
Mycoplasma contamination	Not applicable
Commonly misidentified lines (See ICLAC register)	Not applicable

Plants

Seed stocks	*Report on the source of all seed stocks or other plant material used. If applicable, state the seed stock centre and catalogue number. If plant specimens were collected from the field, describe the collection location, date and sampling procedures.*
Novel plant genotypes	*Describe the methods by which all novel plant genotypes were produced. This includes those generated by transgenic approaches, gene editing, chemical/radiation-based mutagenesis and hybridization. For transgenic lines, describe the transformation method, the number of independent lines analyzed and the generation upon which experiments were performed. For gene-edited lines, describe the editor used, the endogenous sequence targeted for editing, the targeting guide RNA sequence (if applicable) and how the editor was applied.*
Authentication	*Describe any authentication procedures for each seed stock used or novel genotype generated. Describe any experiments used to assess the effect of a mutation and, where applicable, how potential secondary effects (e.g. second site T-DNA insertions, mosiacism, off-target gene editing) were examined.*
