# Supplementary material for: A selectivity filter in Tim17 mediates intramitochondrial protein sorting
Source: Nat Commun. 2026 Oct 7;17:11424. doi: 10.1038/s41467-026-77990-3 (PMC13736910; doi:10.1038/s41467-026-77990-3)
Supplement: Supplementary file 3 [file 41467_2026_77990_MOESM1_ESM.pdf]

Supplementary Information

A selectivity filter in Tim17 mediates intramitochondrial protein sorting

Annika Egeler, Sanjoy Paul, Rosario Valenti, et al.

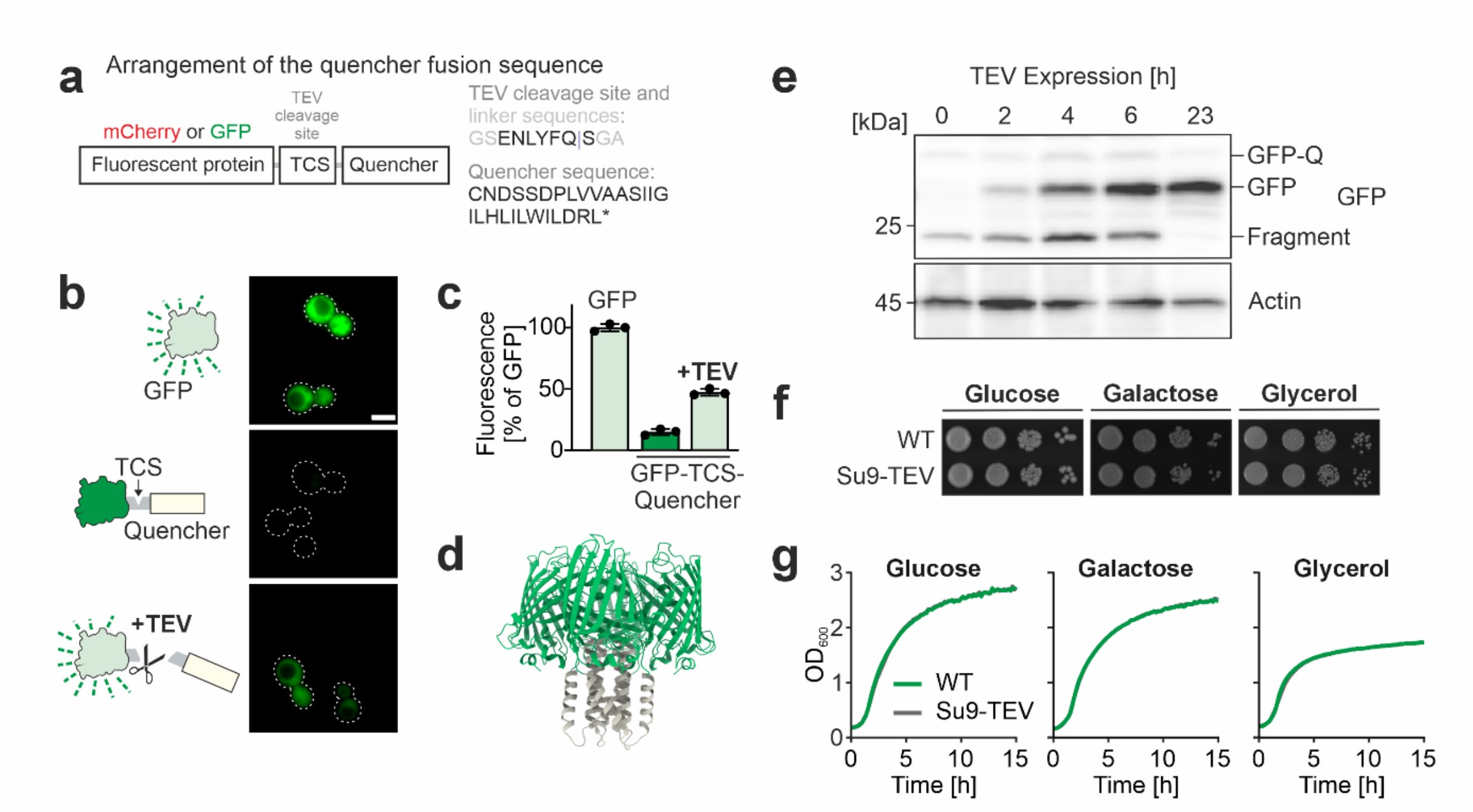

Supplementary Fig. 1. A protein sorting reporter that relies on fluorescence dequenching. a, Schematic representation of the Quencher fusion construct consisting of either GFP or mCherry connected via the TEV cleavage site (TCS) to the Quencher peptide. **b**,**c**, GFP as well as a fusion protein of GFP, TEV cleavage site (TCS) and the quencher domain were expressed in the cytosol of yeast cells using a constitutive *TPI* promoter. For the last sample, the TEV protease was coexpressed with the fusion protein. Fluorescence levels were assessed by microscopy and, in addition, quantified by fluorescence spectrometry. Shown are mean values and standard deviations from three independent measurements. Scale bar = 5 µm. **d**, The GFP-Quencher fusion protein forms a tetramer, resulting in fluorescence quenching. The structure of the complex was modeled using AlphaFold 3 [1]. **e**, The TEV protease was expressed from a *GAL* promoter for the indicated time points. Whole cell extracts were analyzed by Western blotting, with the quencher construct detected before (GFP–Q) and after (GFP) cleavage by TEV. **f**, **g**, Yeast cells carrying either no mitochondrial TEV protease (WT, wild type) or a genomically integrated construct for the constitutive expression of the matrix-targeted TEV protease (Su9-TEV) were grown to mid-log phase. Cell growth was analyzed by a spot assay of serial dilutions (f), or by measuring optical densities at 600 nm [OD_{600}] in a plate reader (g).

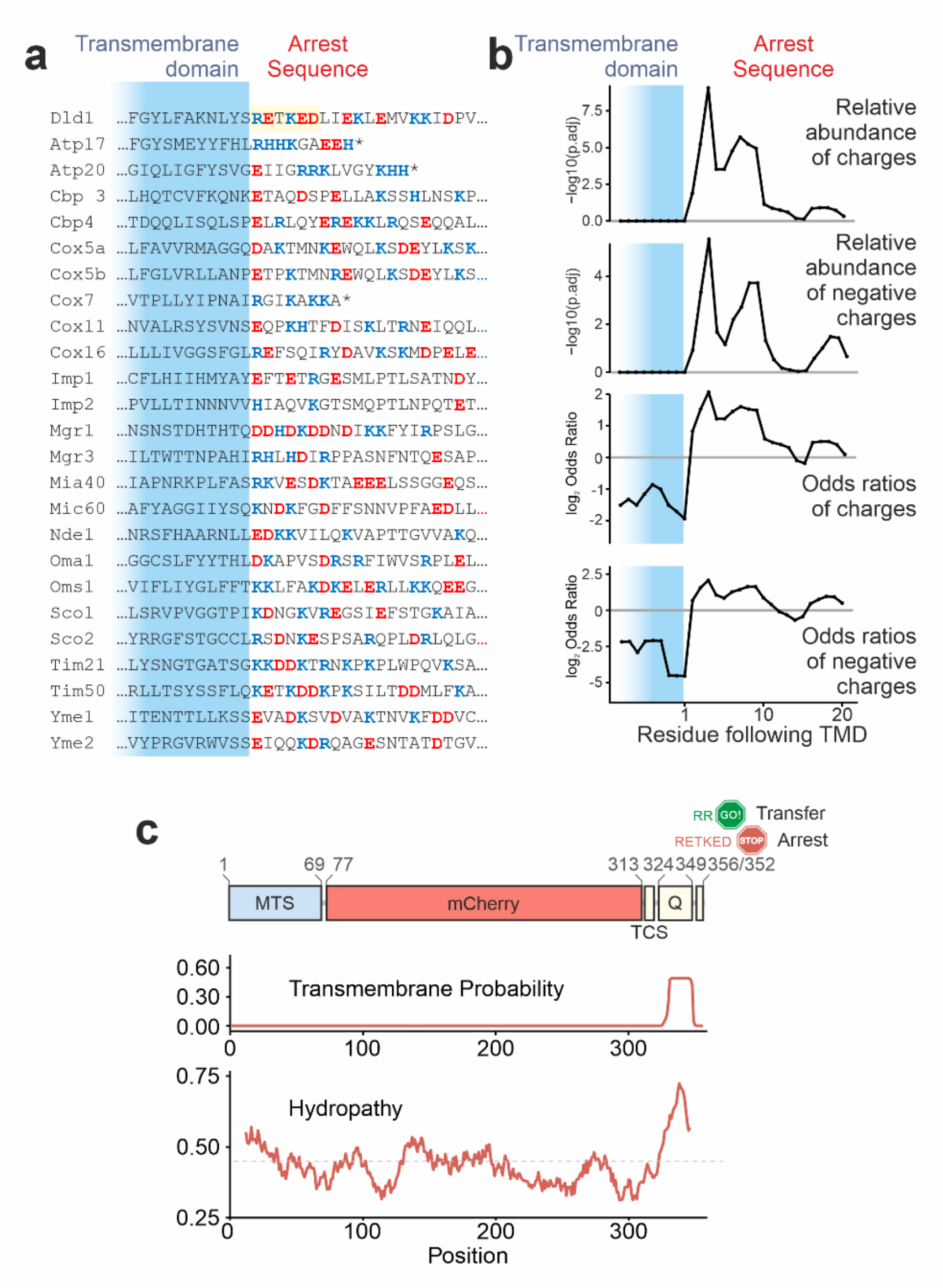

Supplementary Fig. 2. The FLASH reporter uses arrest signals that are common in IM proteins. a, Sequences of mitochondrial IM proteins with an N(in)-C(out) topology and an N-terminal MTS are shown. The C-terminal segment of the TMD and of the initial IMS-exposed residues are shown. Charged residues are indicated. **b**, Charged, in particularly negatively charged amino acid residues are over-represented after TMDs. Using a moving window of 3 residues, charged (negatively charged) and uncharged residues were counted within each window and compared against the counts obtained from the entire yeast proteome. Odds ratios and p-values were calculated using a one-sided Fisher's exact test for each window. P-values were adjusted using the Benjamini-Yekutieli procedure. **c**, Schematic representation of the FLASH sensor and its predicted transmembrane probability and hydropathy profiles. The FLASH sensor contains the Su9 mitochondrial targeting sequence (MTS), mCherry, the TEV cleavage site (TCS), and the quencher domain (Q), followed either by the Arrest sequence (RETKED) or the Transfer sequence (RR). Numbers indicate amino acid residue positions within the construct. Transmembrane probability was predicted using TMHMM 2.0 [2], and hydropathy scores were calculated using a 21-residue sliding window [3].

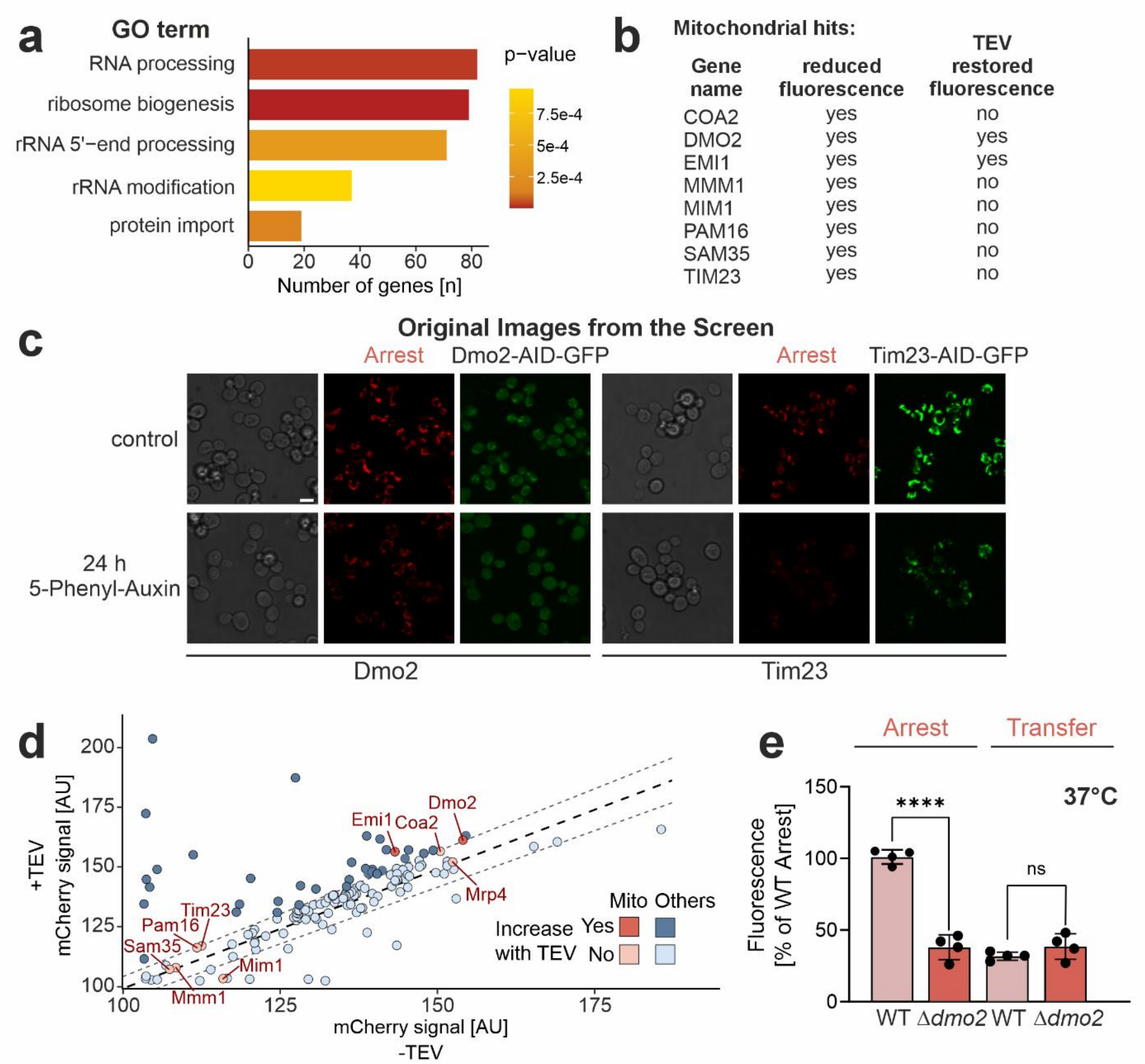

Supplementary Fig. 3. Mutants lacking Dmo2 show reduced signals with the FLASH Arrest reporter. **a**, Proteins for which the auxin-induce depletion diminished the fluorescence of the Arrest reporter (lowest 10%) were analyzed by GO term analysis. Since low gene expression also leads to low expression of the Arrest reporter, components of the transcription and translation systems were enriched here. **b**, Mitochondrial proteins whose depletion resulted in low fluorescence of the Arrest reporter. **c**, Shown are the original images from the screen exemplarily for the strains in which Dmo2 and Tim23 were tagged with the AID-GFP cassette. Scale bar = 5 µm. **d**, The screen was carried out the presence or absence of a genomically integrated cassette for the expression of mitochondria-targeted TEV. The fluorescence signals of the Arrest reporter were quantified from microscopy images. Please note that the presence of TEV mitigates the fluorescence loss caused by the depletion of Dmo2 (and Emi1). A 5% deviation from the diagonal was used as a cut-off. **e**, The FLASH reporter signals in the indicated strains grown at 37°C were quantified in a plate-operated fluorescence spectrometer. Shown are mean values and standard deviations of 3 independent replicates. Statistical differences were calculated using a one-way ANOVA. **** Adjusted *P* <0.0001, ns adjusted *P* = 0.5239.

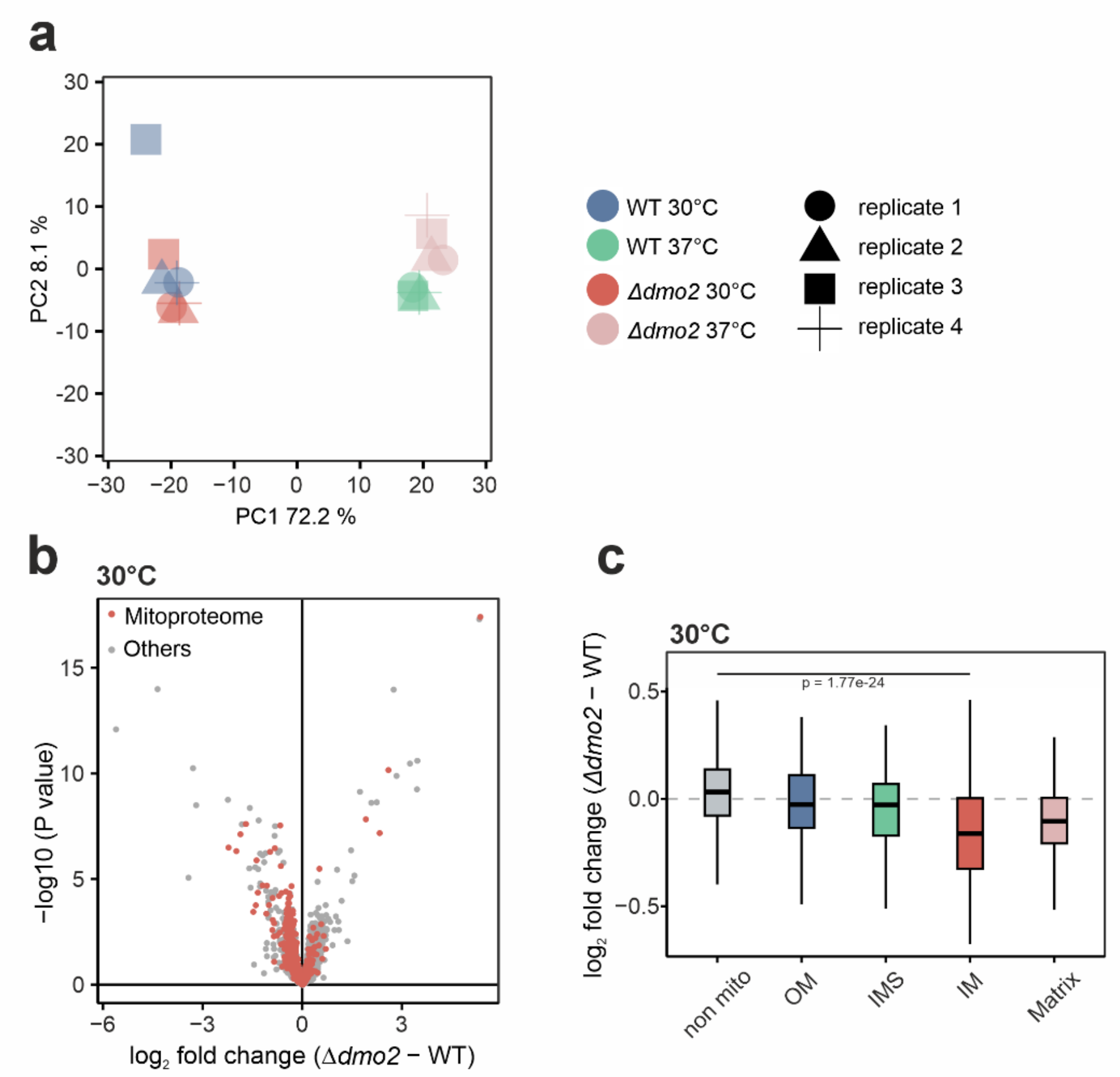

Supplementary Fig. 4. The absence of Dmo2 leads to the depletion of IM proteins. a, Principal component analysis of the whole cell proteomes of the indicated strains. **b**, **c**, Wild type and *Δdmo2* cells were grown in galactose-based medium at 30 and 37°C, lysed and subjected to mass spectrometry. Four independent biological replicates were used for each sample. The data correspond to those shown in Fig. 2j and k, but here the values for the cultures grown at 30 °C are shown. Significance was calculated by a two-sided Wilcoxon rank sum test with continuity correction (Benjamini-Hochberg).

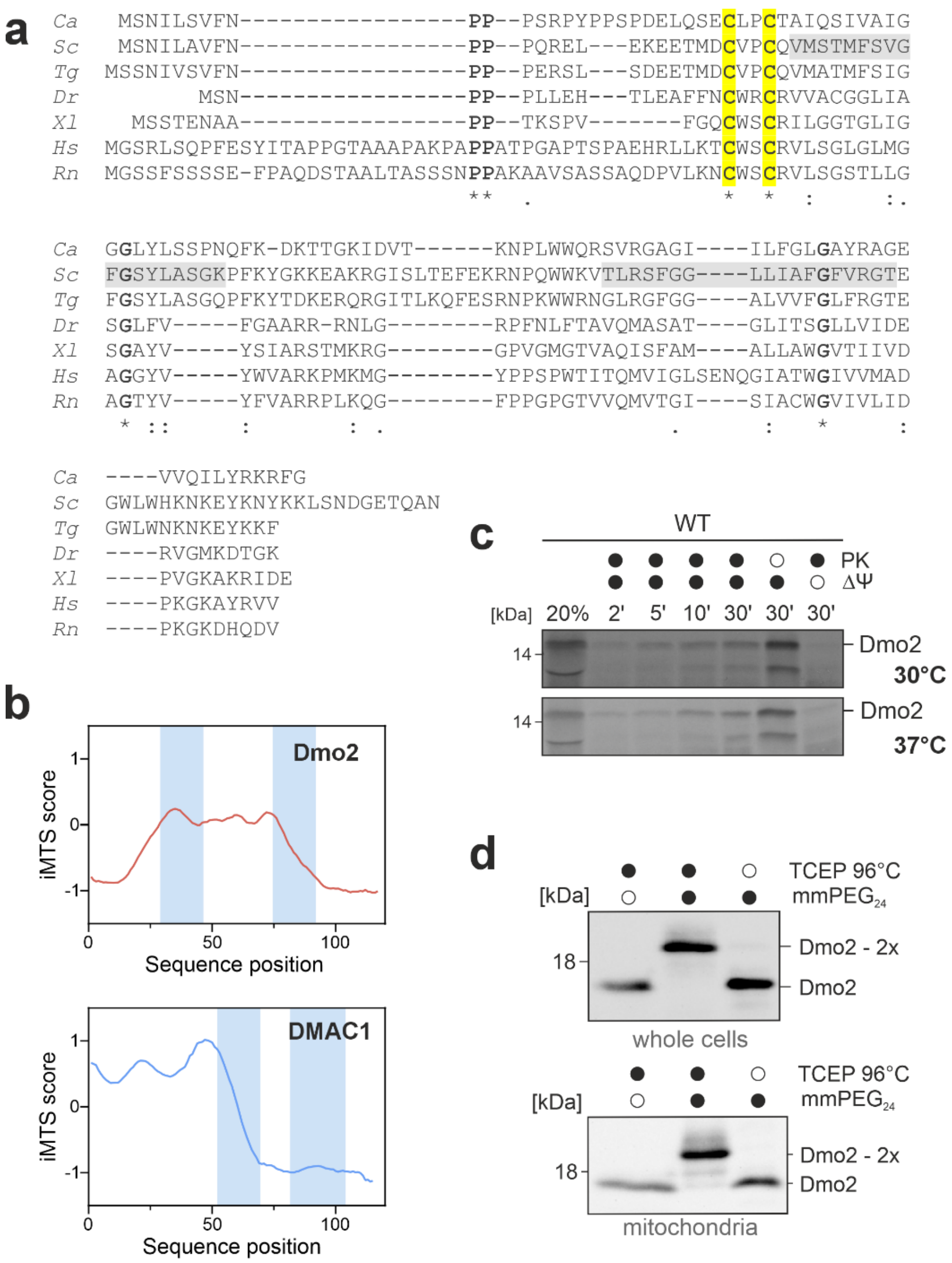

Supplementary Fig. 5. Dmo2 is a conserved oxidoreductase. a, The sequences of Dmo2 and its homologs were aligned using the Muscle alignment tool [4]. The following sequences were used: *Ca*, *Candida albicans* XP_715135.1; *Sc*, *Saccharomyces cerevisiae* NP_010124.1; *Tg*, *Torulaspora globosa* XP_037138321.1; *Dr*, *Danio rerio* AAI62207.1; *Xl*, *Xenopus laevis* XP_018113160.1; *Hs*, *Homo sapiens* sp|Q96GE9; *Rn*, *Rattus norvegicus* NP_001193604.1. The two conserved cysteine residues are highlighted. **b**, Shown are the iMTS scores predicted for the sequences of Dmo2 and human DMAC1 (http://iMLP.bio.uni-kl.de) [5,6]. The positions of the TMDs in the proteins are indicated. **c**, Radiolabeled Dmo2 was incubated at 30 and 37°C with isolated mitochondria for the times indicated. Non-imported protein was removed by proteinase K (PK). In one sample, the membrane potential (Δψ) was dissipated before Dmo2 was added. **d**, Dmo2 was expressed in yeast cells with a C-terminal hemagglutinin tag (HA). Lysed cells and isolated mitochondria were treated with 15 mM $mmPEG_{24}$ in the presence or absence of 5 mM TCEP. Proteins were subjected to SDS-PAGE and Western blotting with antibodies against the HA-tag.

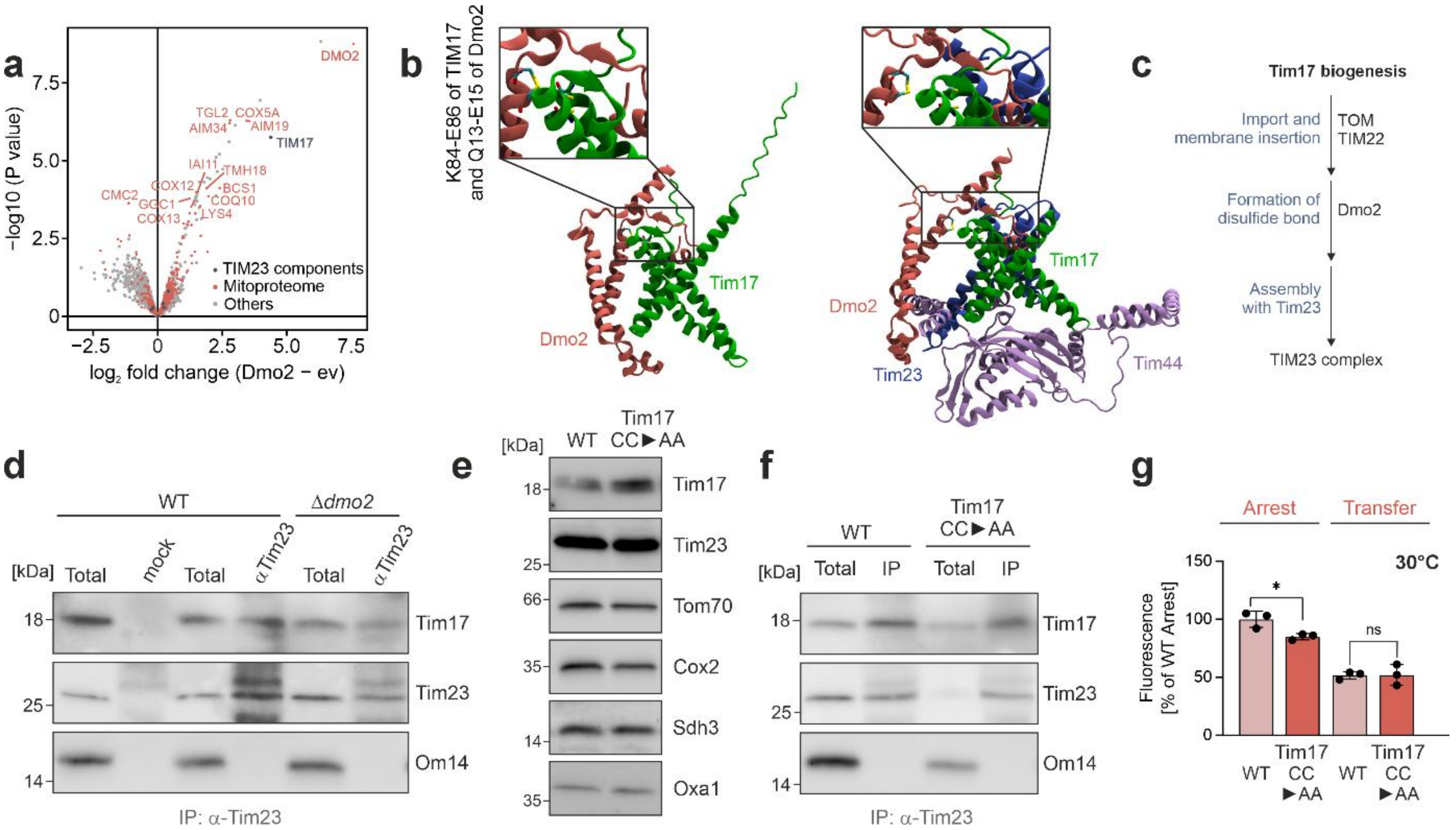

Supplementary Fig. 6. Dmo2 introduces a disulfide bond into Tim17 that is important for the correct insertion of IM proteins. a, Volcano plot to visualize the proteins co-isolated with Dmo2-HA. Refers to Fig. 4a. **b**, The Alphafold structure of Dmo2 was modeled onto the structure of the Tim17–Tim23–Tim44 complex. Please note the overlap of Dmo2 with Tim23 that suggests that the binding of these proteins to Tim17 is mutually exclusive. **c**, Proposed sequence of events during Tim17 biogenesis. **d**, Wild type and *Δdmo2* mitochondria were lysed with 0,6% digitonin. The extract was either directly loaded (Total, corresponding to 5%) or used for an immunoprecipitation with Tim23-specific antibodies. For a mock control sample, antibodies were omitted. Please note that Tim17 was efficiently coisolated with Tim23 in both wild type and *Δdmo2* mitochondria. **e**, Whole cell extracts of the indicated strains were analyzed by Western blotting. **f**, Tim23 immunoprecipitates were analyzed as described for d. **g**, The FLASH reporters were transformed into wild type and Tim17C10,77A mutant cells. The fluorescence intensity was quantified by fluorescence spectrometry. Shown are mean values and standard deviations from three independent measurements. Statistical differences were calculated using a one-way ANOVA. * Adjusted $P = 0.0483$.

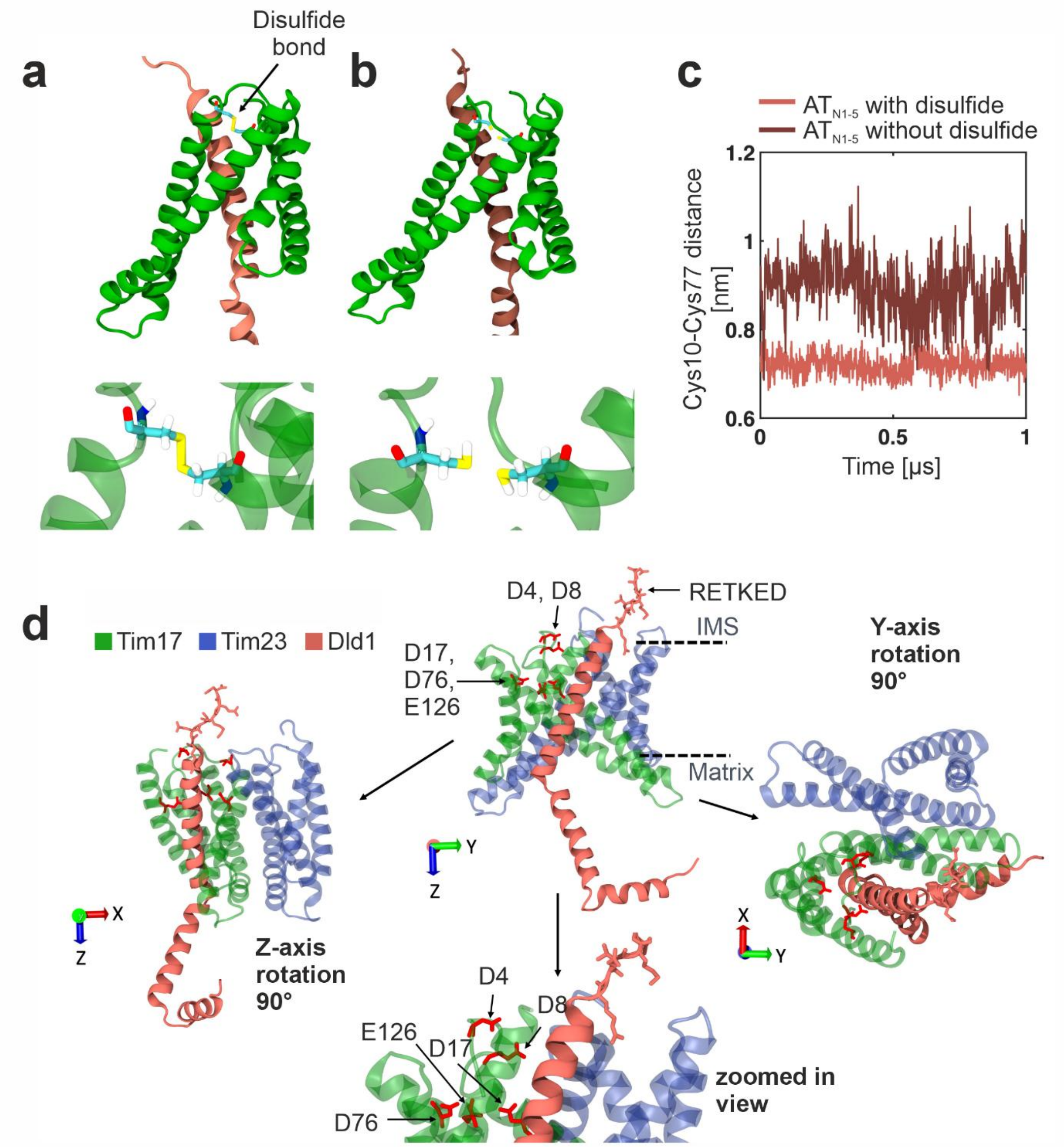

Supplementary Fig. 7. The disulfide bond in Tim17 is important for the stability and positioning of charged residues in the N-terminus of Tim17. a,**b**, Tim17 and Dld1 are shown in cartoon representation for (a) the intact and (b) the broken disulfide bond states. Enlarged views of the constituent cysteine residues (Cys10 and Cys77) are shown below each structure. Here, hydrogen atoms of cysteine residues are also shown. **c**, Time evolution (see Video S2) of the distance between the backbone atoms of Cys10 and Cys77 during the simulation. **d**, Cartoon representations of Dld1, Tim17, and Tim23, with Tim17 residues D4, D8, D17, D76, and E126 highlighted as red sticks. D4 and D8 are oriented toward the IMS, while D17, D76, and E126 are positioned near the transmembrane core. The RETKED motif of Dld1 is also shown as sticks. Two orientations of the complex are presented: rotated 90° along the Z-axis (left) and along the Y-axis (right). Enlarged views below highlight the negatively charged residues of Tim17 and the RETKED motif of Dld1.

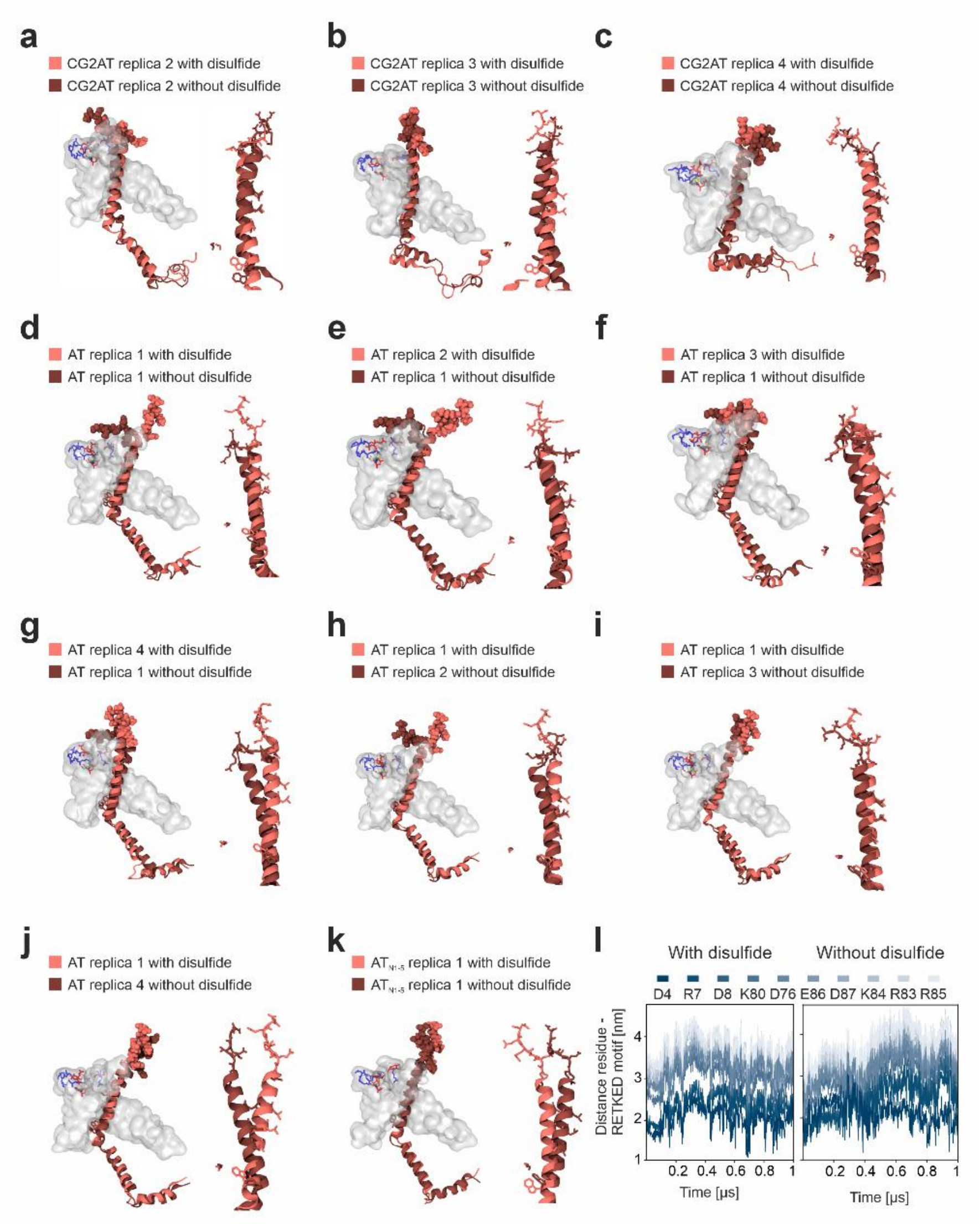

Supplementary Fig. 8. Snapshots of Dld1 in the presence and absence of the disulfide bond in Tim17 across different replica simulations. Tim17 is shown as a transparent surface with its IMS facing charged residue (red: acidic, blue: basic) cluster, Cys10, and Cys77 in stick representation. Dld1 is shown in cartoon representation while its key residues (W43, S51, L55, L59) are depicted as sticks to illustrate their vertical positioning within the Tim17 cavity, both with and without the disulfide bond. G29 of Tim17 is included as a positional reference. Structural alignment was performed with respect to Tim17 to enable accurate comparison of Dld1 positioning. **a**-**c**, Replicas 2–4 of the CG2AT model, each initiated from distinct time points of the coarse-grained trajectory; replica 1 is shown in Fig. 5b–d. In total, four AT model replicas were run from identical starting coordinates and velocities. **d**-**g**, All four replicas of the AT model with the disulfide bond compared to replica 1 without the disulfide bond. **h**-**j**, Replicas 2–4 of the AT model without the disulfide bond compared to replica 1 with the bond. **k**, Corresponding snapshots for the $AT_{N1–5}$ model. **l**, Distances between charged residues of Tim17 (D4, R7, D8, D76, K80, R83, K84, R85, E86, D87) and the RETKED motif of Dld1 in the presence and absence of the disulfide bond in the $AT_{N1–5}$ model. For distance calculations all atoms were considered except hydrogen atoms. The residues shown were ordered by increasing distance to the RETKED arrest signal of Dld1.

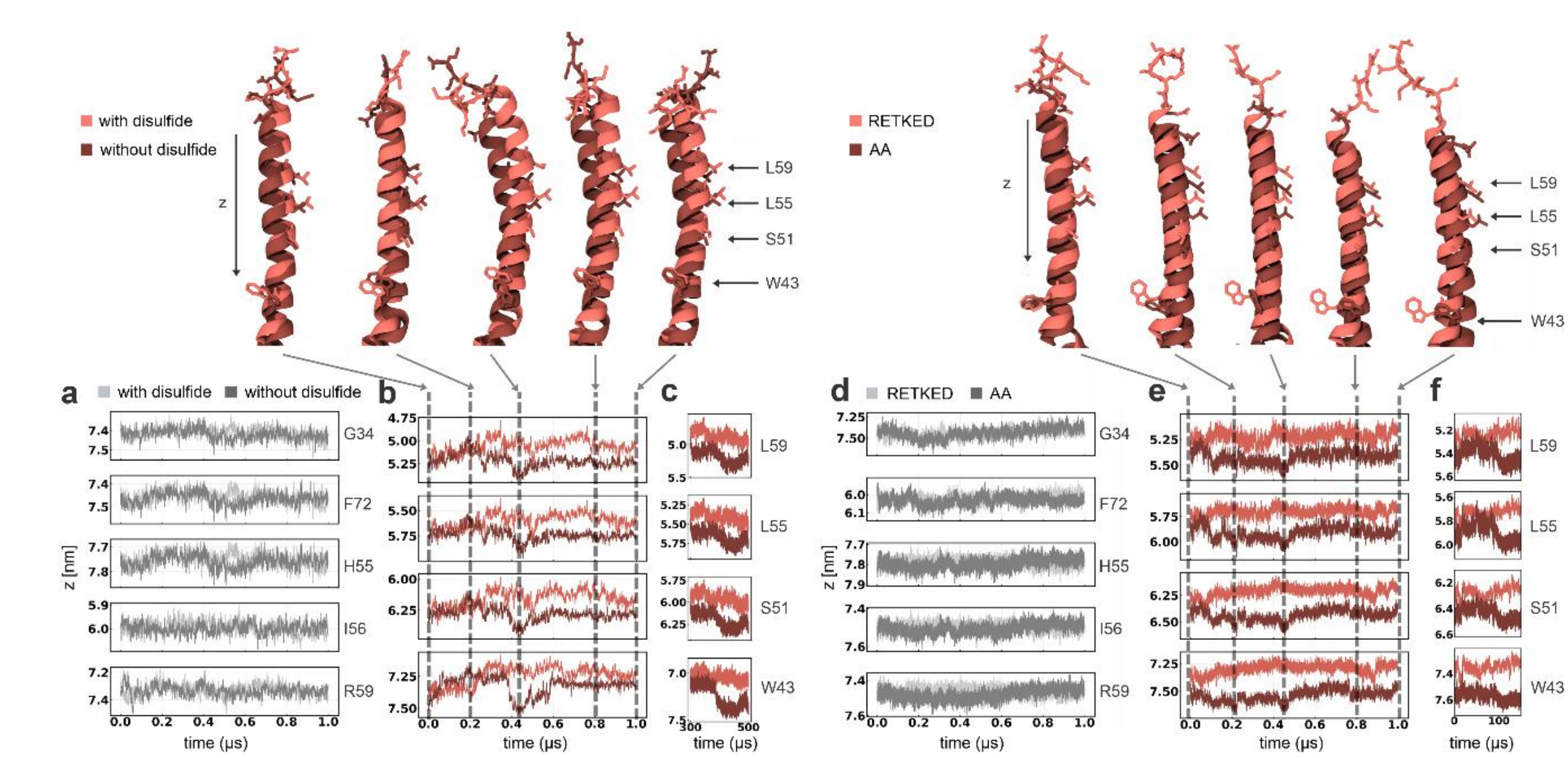

Supplementary Fig. 9. Comparative translocation dynamics of Dld1 through the Tim17 cavity. a, Z-coordinate positions of selected residues from Tim17 (G34, F72) and Tim23 (H55, I56, R59) over time, shown as a reference. The vertical position of the Tim17/Tim23 cavity remains stable regardless of the presence or absence of the disulfide bond in Tim17. **b**, Time evolution of the Z coordinates of substrate residues L59, L55, S51 and W43 reveals a pronounced downward displacement when the Tim17 disulfide bond is absent compared with the disulfide-bonded state. This downward movement becomes more pronounced after around 400 ns of the simulation. **c**, Zoomed-in view on the Z-coordinate positions of the substrate residues between 300 and 500 ns. **d**, **e**, Same analysis as shown in a, b, comparing the vertical positions of Dld1 containing either the arrest signal (RETKED) or the transfer signal (AA). **c**, Zoomed-in view on the first 150 ns of the Z-coordinate positions of the substrate residues.

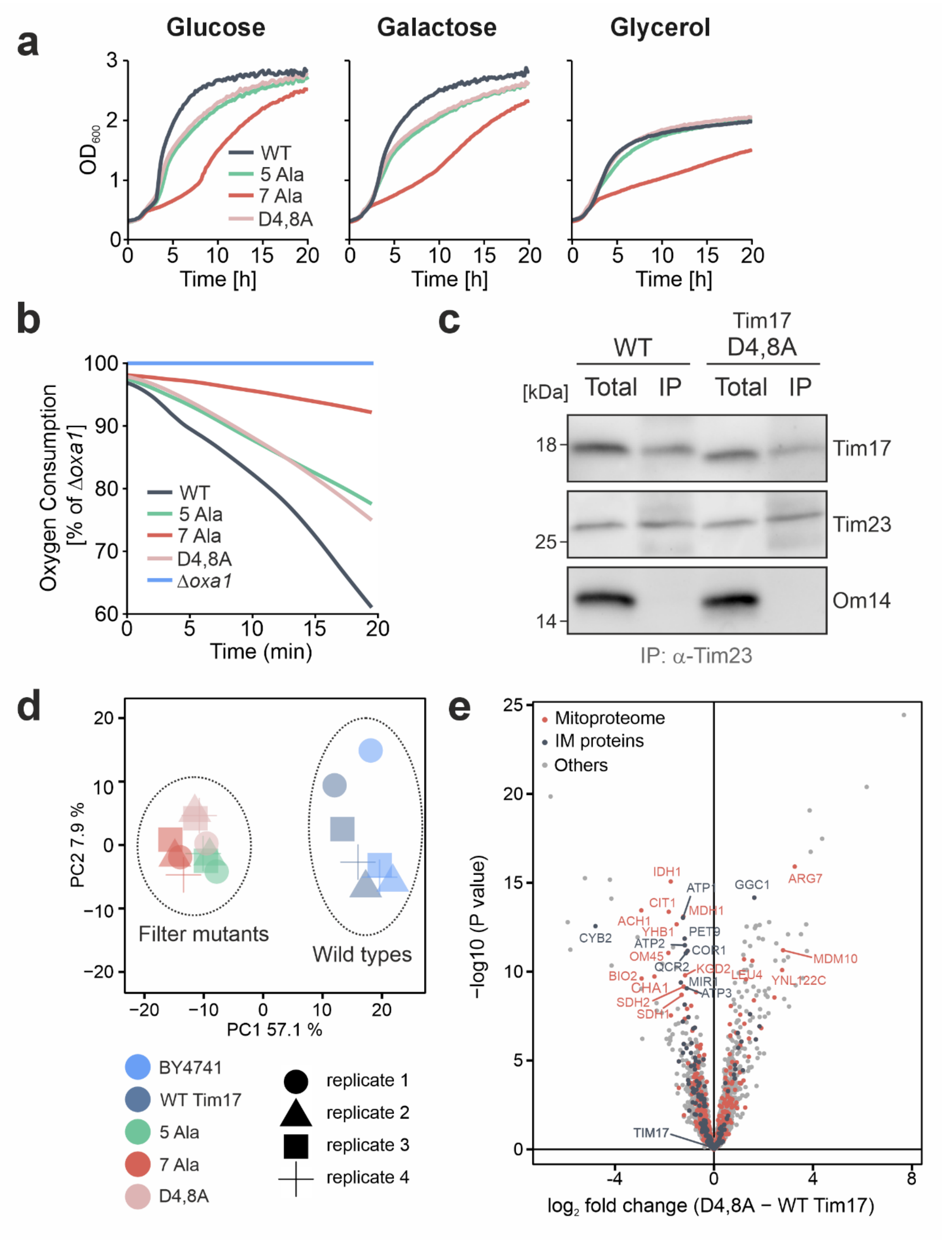

Supplementary Fig. 10. The selectivity filter in Tim17 is of physiological relevance. a, The indicated strains were grown to log phase in galactose medium, sedimented, washed and adjusted to OD 0.1. Growth in a plate reader under agitation was continuously monitored over time at 30°C. **b**, Oxygen consumption was measured in the indicated strains over time. The respiration-deficient *Δoxa1* mutant was used for control. **c**, Tim23 immunoprecipitates were analyzed as described for Supplementary Fig. 4d. **d**, **e**. Whole cell proteomes of the indicated strains were analyzed as described for Fig. 2i-k. These data belong to the graphs shown in Fig. 6i and j.

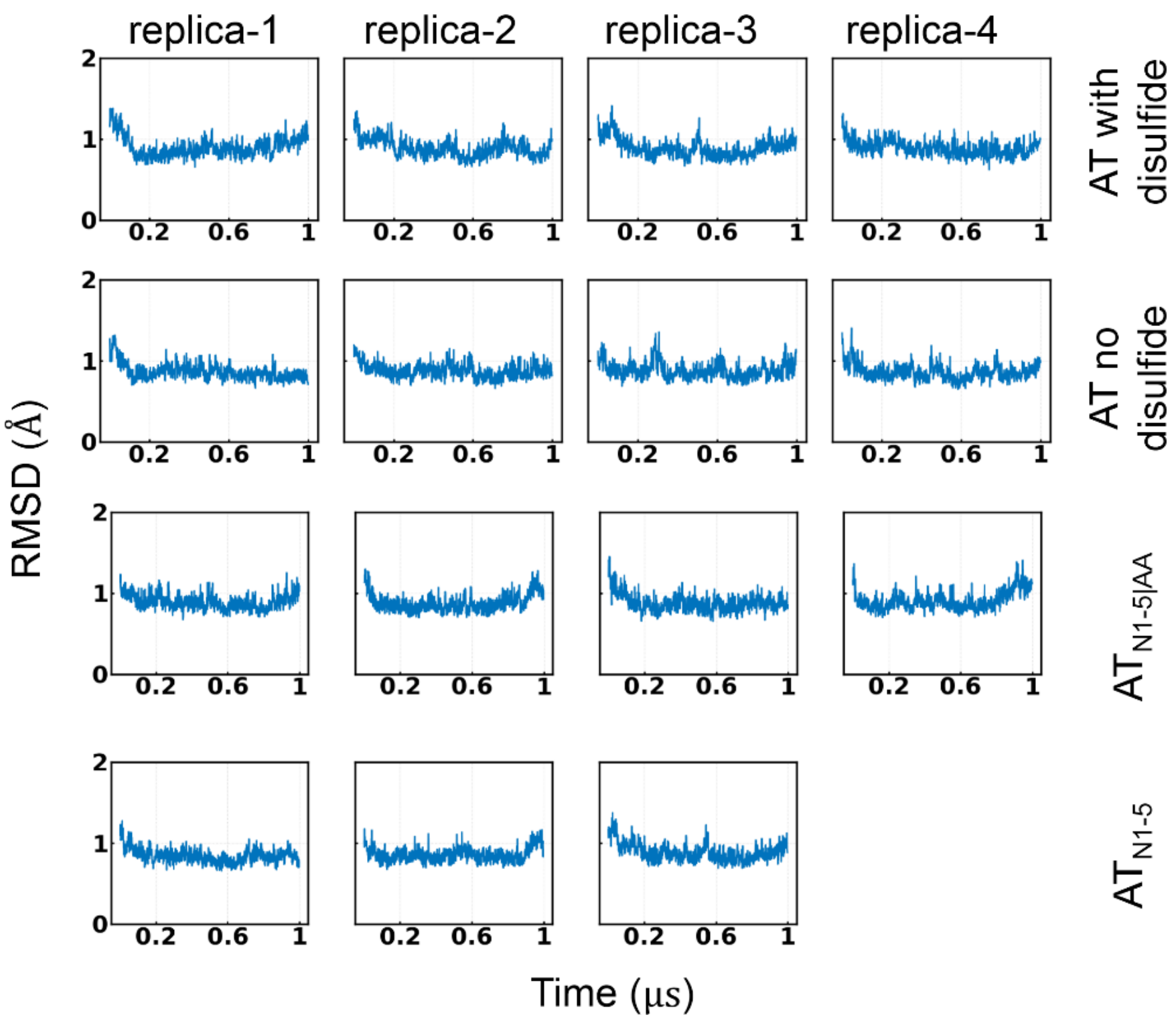

Supplementary Fig. 11. Convergence of backbone RMSD trajectories in rigid residues of Tim17 and Tim23 across TIM23 complex models. The root mean square deviation (RMSD) evolution of the backbone atoms in the rigid residues of Tim17 and Tim23 within the TIM23 complex reaches convergence within the 1 μs timescale across different replicas of the AT with disulfide, AT without disulfide, ATN1-5|AA, and ATN1-5 models.

	AT with disulfide	ATN1-5|AA	ATN1-5	CG2AT with disulfide
DOPC	209	228	228	239
DOPE	132	144	144	150
DOPS	22	24	24	24
POPI	88	96	96	100
TOCL2	88	96	96	100
POPA	11	12	12	11
Water	63662	61687	67554	110227
Na^+	443	466	483	704
Cl^-	174	170	186	398

Supplementary Table 1. System composition (number of molecules) for atomistic MD simulation.

	Condition	Integration time step (fs)	Simulation length (ps)	k backbone	k sidechain	k lipids	k dihedrals
				kJ mol^{-1} nm^{-2}	kJ mol^{-1} nm^{-2}	kJ mol^{-1} nm^{-2}	kJ mol^{-1} nm^{-2}
1	NVT	1	125	4000	2000	1000	1000
2	NVT	1	125	2000	1000	400	400
3	NpT	1	125	1000	500	400	200
4	NpT	2	500	500	200	200	200
5	NpT	2	500	200	50	40	100
6	NpT	2	500	50	0	0	0
7	NpT	2	10000	0	0	0	0

Supplementary Table 2. Conditions for equilibration steps performed before production run during atomistic MD simulations.

Figure	Code
Fig. 5a-c	VMD-1.9.4
Fig. 5d-e	gmx density
Suppl. Fig. 7a-d	VMD-1.9.4
Suppl. Fig. 8a-l	VMD-1.9.4
Suppl. Fig. 9a-f	MDAnalysis
Suppl. Fig. 11	MDAnalysis

Supplementary Table 3.| Code used for MD simulation data analysis and visualization.

References

1 Abramson, J. *et al.* Accurate structure prediction of biomolecular interactions with AlphaFold 3. *Nature* **630**, 493-500 (2024).
2 Krogh, A., Larsson, B., von Heijne, G. & Sonnhammer, E. L. Predicting transmembrane protein topology with a hidden Markov model: application to complete genomes. *J. Mol. Biol.* **305**, 567-580 (2001).
3 Kyte, J. & Doolittle, R. F. A simple method for displaying the hydropathic character of a protein. *J. Mol. Biol.* **157**, 105-132 (1982).
4 Madeira, F. *et al.* The EMBL-EBI Job Dispatcher sequence analysis tools framework in 2024. *Nucleic Acids Res* **52**, W521-W525 (2024).
5 Schneider, K., Zimmer, D., Nielsen, H., Herrmann, J. M. & Mühlhaus, T. iMLP, a predictor for internal matrix targeting-like sequences in mitochondrial proteins. *Biol Chem* **402**, 937-943 (2021).
6 Jung, F., Rödl, S., Herrmann, J. M. & Muhlhaus, T. Analysis and prediction of internal mitochondrial targeting signals. *Methods Enzymol* **706**, 263-283 (2024).
