# Supplementary material for: A selectivity filter in Tim17 mediates intramitochondrial protein sorting
Source: Nat Commun. 2026 Oct 7;17:11424. doi: 10.1038/s41467-026-77990-3 (PMC13736910; doi:10.1038/s41467-026-77990-3)
Supplement: Supplementary file 4 [file 41467_2026_77990_MOESM2_ESM.pdf]

Description of Additional Supplementary Files

File Name: Supplementary Video 1

Snapshot of the TIM23 complex (color scheme as in Fig. 5A) embedded in the membrane after 1 μs of atomistic MD simulation. The RETKED motif of the Dld1 substrate is shown as van der Waals spheres, the disulfide bond in Tim17 is depicted as sticks, and residues D4 and D8 of Tim17 are highlighted in red stick representation.

File Name: Supplementary Video 2

Dynamics of Tim17 helices in the presence (left) and absence (right) of the disulfide bond.

File Name: Supplementary Video 3

Dynamics of Dld1 within the Tim17 cavity, with and without the disulfide bond (stick representation) in Tim17, from replica 1 simulation of the CG2AT model (as shown in Fig. 5C and D). To facilitate precise comparison of Dld1 positioning, structural alignment was carried out with respect to Tim17. For clarity, Tim17 is shown as a transparent surface. Dld1 residues depicted as sticks highlight its downward displacement toward the matrix in the absence of the disulfide bond.

File Name: Supplementary Video 4

Comparison of Dld1 dynamics in the wild-type form and the RETKED-to-alanine (AA) mutant (as shown in Fig-5E), with the disulfide bond in Tim17 intact in both cases. The RETKED motif and its AA variant are shown as sticks, while Tim17 is depicted as a transparent surface for clarity. The RETKED-to-AA mutation leads to a vertical downward shift of Dld1.

File Name: Supplementary Video 5

Dynamics of Dld1 in AT vs. AT_{N1-5} model (shown in Fig-6A-B). D4 and D8 (red stick) of AT_{N1-5} model, serving as the crucial player for precise positioning of Dld1, is represented as red sticks.

File Name: Supplementary Data 1

Strains used in this study

File Name: Supplementary Data 2

Plasmids used in this study

File Name: Supplementary Data 3

Results of the genetic screen using the FLASH assay

File Name: Supplementary Data 4

Mass spectrometry data of whole cell proteomes of *Ddmo2* and wild type cells

File Name: Supplementary Data 5

Mass spectrometry data of Dmo2 pulldown experiments

File Name: Supplementary Data 6

Mass spectrometry data of whole cell proteomes of Tim17 selectivity filter mutants

File Name: Supplementary Data 7

Light microscopy reporting table for our study
