# Supplementary material for: A selectivity filter in Tim17 mediates intramitochondrial protein sorting
Source: Nat Commun. 2026 Oct 7;17:11424. doi: 10.1038/s41467-026-77990-3 (PMC13736910; doi:10.1038/s41467-026-77990-3)
Supplement: Supplementary file 5 [file 41467_2026_77990_MOESM17_ESM.zip › Source Data/Source Data Fig 1.pdf]

Figure 1

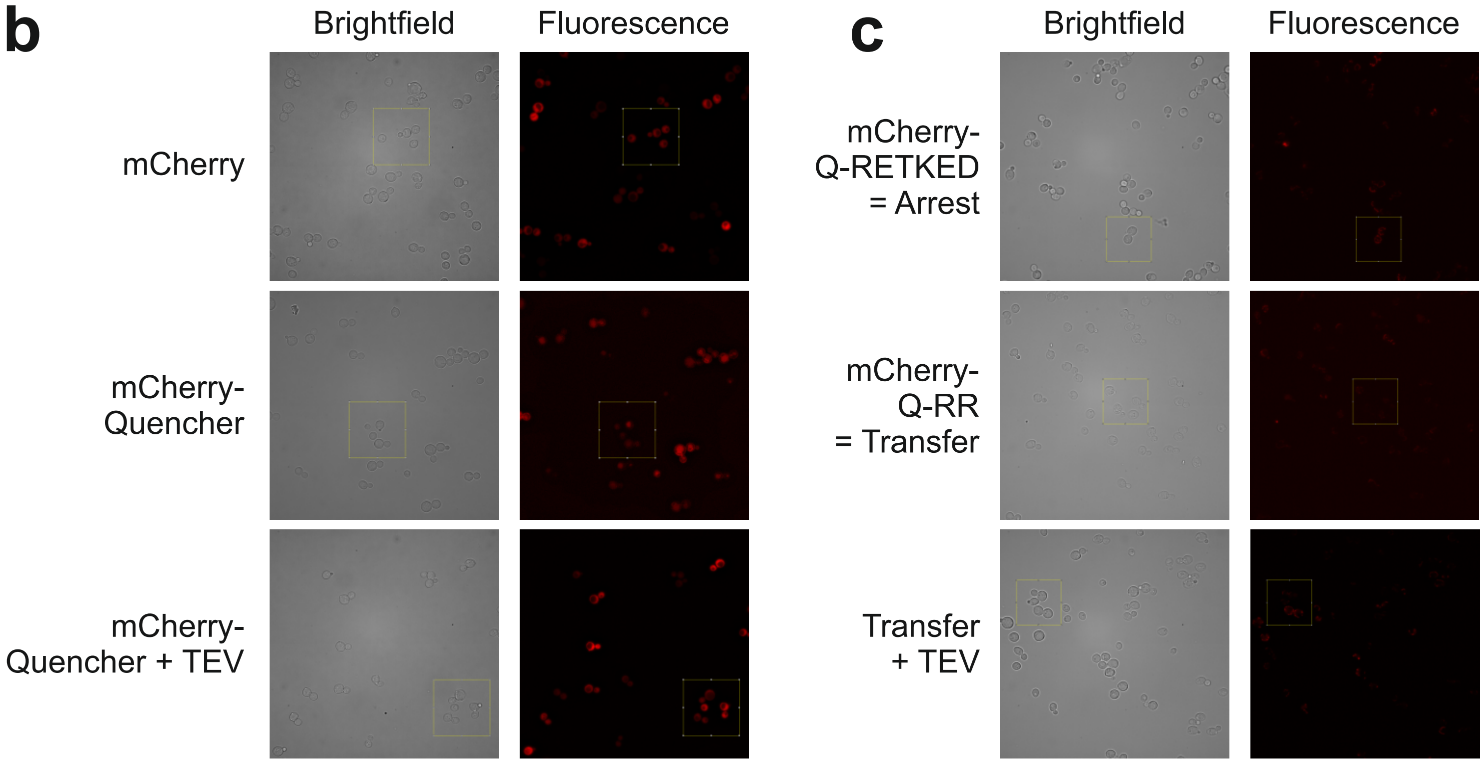
b
Brightfield
Fluorescence
mCherry
mCherry-
Quencher
mCherry-
Quencher + TEV
c
Brightfield
Fluorescence
mCherry-
Q-RETKED
= Arrest
mCherry-
Q-RR
= Transfer
Transfer
+ TEV

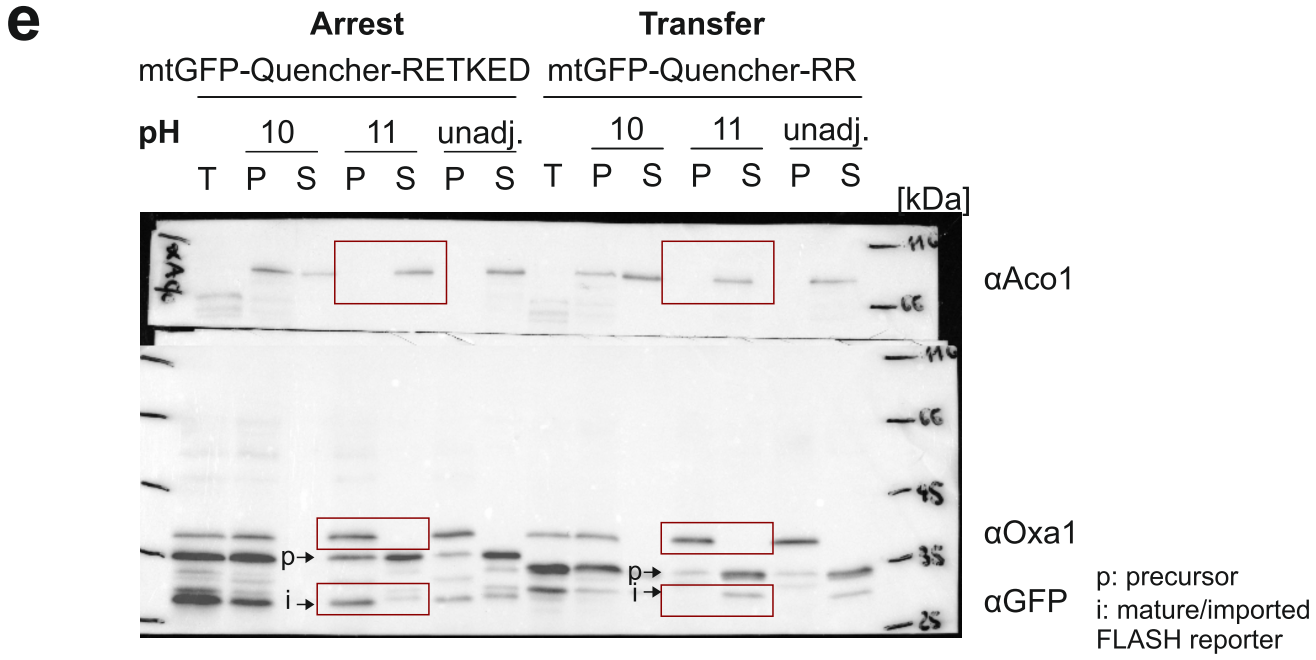
e
Arrest
mtGFP-Quencher-RETKED
Transfer
mtGFP-Quencher-RR
pH
10
11
unadj.
10
11
unadj.
T P S P S P S T P S P S P S
[kDa]
αAco1
αOxa1
αGFP
p
i
p: precursor
i: mature/imported
FLASH reporter
