# Supplementary material for: A selectivity filter in Tim17 mediates intramitochondrial protein sorting
Source: Nat Commun. 2026 Oct 7;17:11424. doi: 10.1038/s41467-026-77990-3 (PMC13736910; doi:10.1038/s41467-026-77990-3)
Supplement: Supplementary file 5 [file 41467_2026_77990_MOESM17_ESM.zip › Source Data/Source Data Fig 2.pdf]

Figure 2

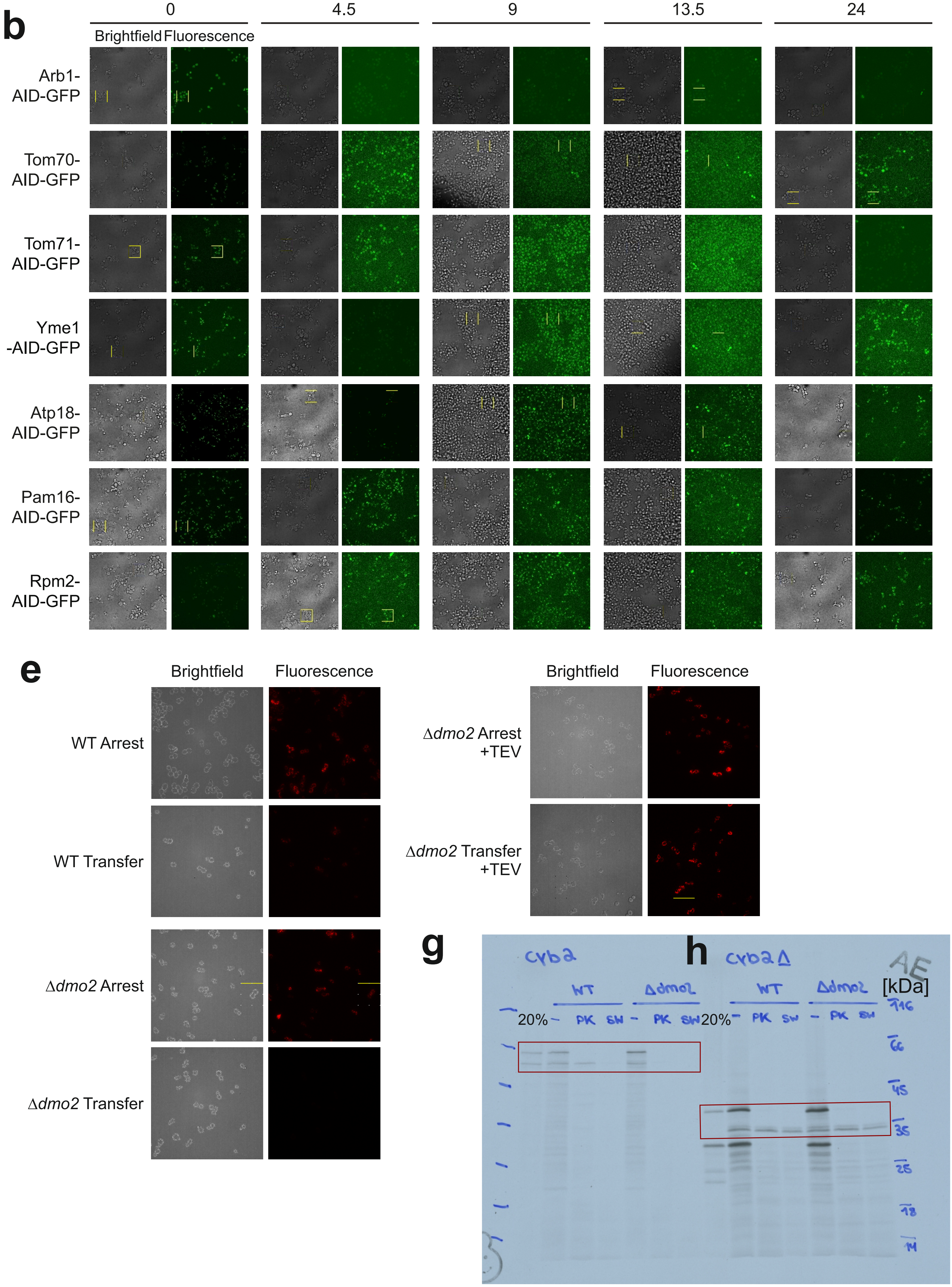
b
0
4.5
9
13.5
24
Brightfield
Fluorescence
Arb1-AID-GFP
Tom70-AID-GFP
Tom71-AID-GFP
Yme1-AID-GFP
Atp18-AID-GFP
Pam16-AID-GFP
Rpm2-AID-GFP
e
Brightfield
Fluorescence
WT Arrest
WT Transfer
Δdmo2 Arrest
Δdmo2 Transfer
Δdmo2 Arrest +TEV
Δdmo2 Transfer +TEV
g
h
Cyb2
Cyb2Δ
WT
Δdmo2
20%
-
PK
SW
[kDa]
116
66
45
35
25
18
14
