# Supplementary material for: A selectivity filter in Tim17 mediates intramitochondrial protein sorting
Source: Nat Commun. 2026 Oct 7;17:11424. doi: 10.1038/s41467-026-77990-3 (PMC13736910; doi:10.1038/s41467-026-77990-3)
Supplement: Supplementary file 5 [file 41467_2026_77990_MOESM17_ESM.zip › Source Data/Source Data Fig 3.pdf]

Figure 3

c

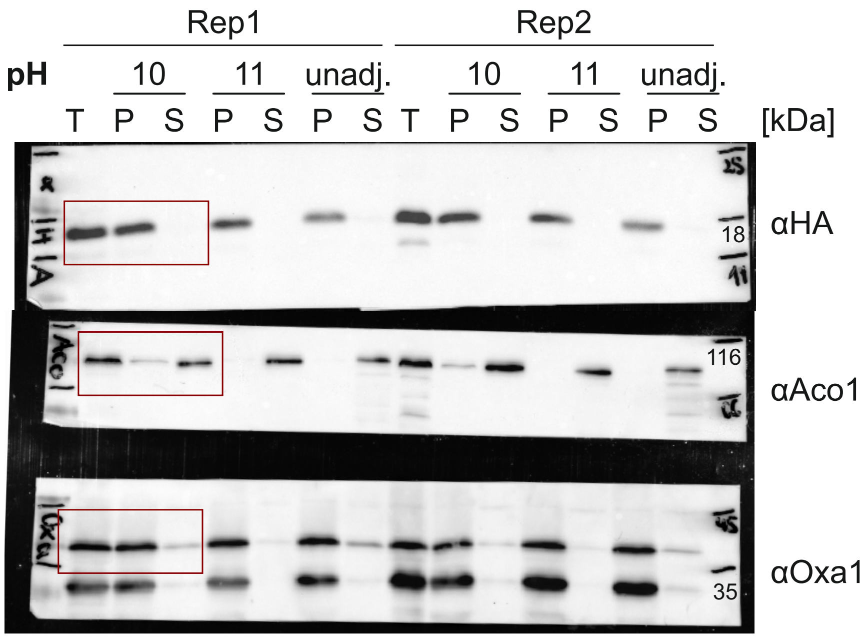
Rep1
Rep2
pH
10
11
unadj.
T P S P S P S T P S P S P S
[kDa]
αHA
18
αAco1
116
αOxa1
35

e

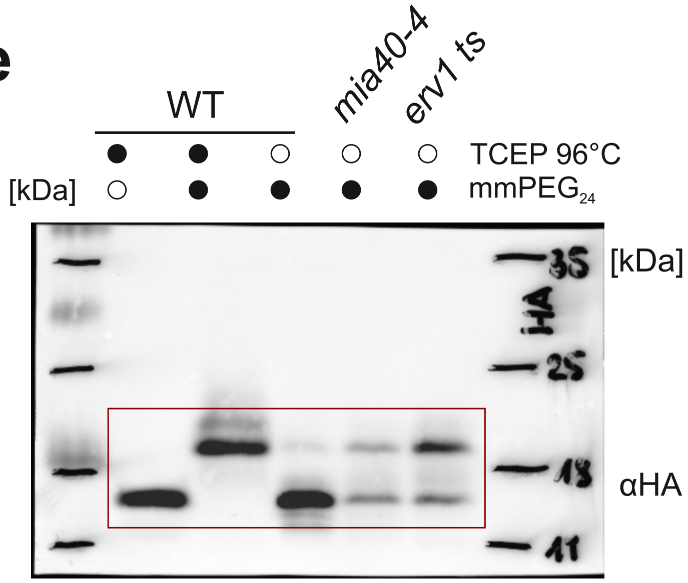
WT
mia40-4
erv1 ts
TCEP 96°C
[kDa]
mmPEG24
[kDa]
αHA

g

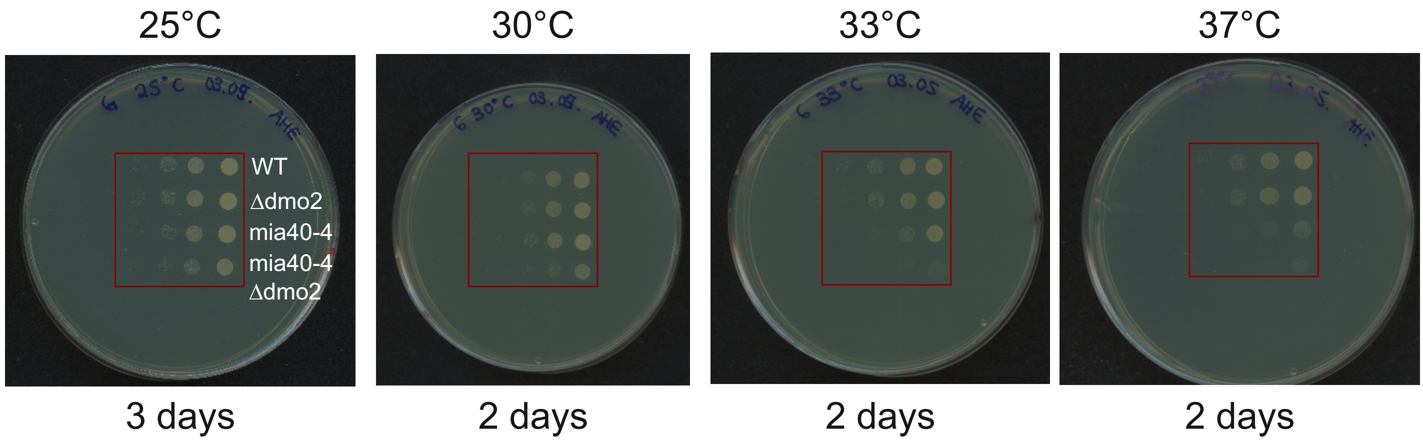
25°C
30°C
33°C
37°C
WT
Δdmo2
mia40-4
mia40-4 Δdmo2
3 days
2 days
2 days
2 days
