# Supplementary material for: A selectivity filter in Tim17 mediates intramitochondrial protein sorting
Source: Nat Commun. 2026 Oct 7;17:11424. doi: 10.1038/s41467-026-77990-3 (PMC13736910; doi:10.1038/s41467-026-77990-3)
Supplement: Supplementary file 5 [file 41467_2026_77990_MOESM17_ESM.zip › Source Data/Source Data Fig 4.pdf]

Figure 4

b

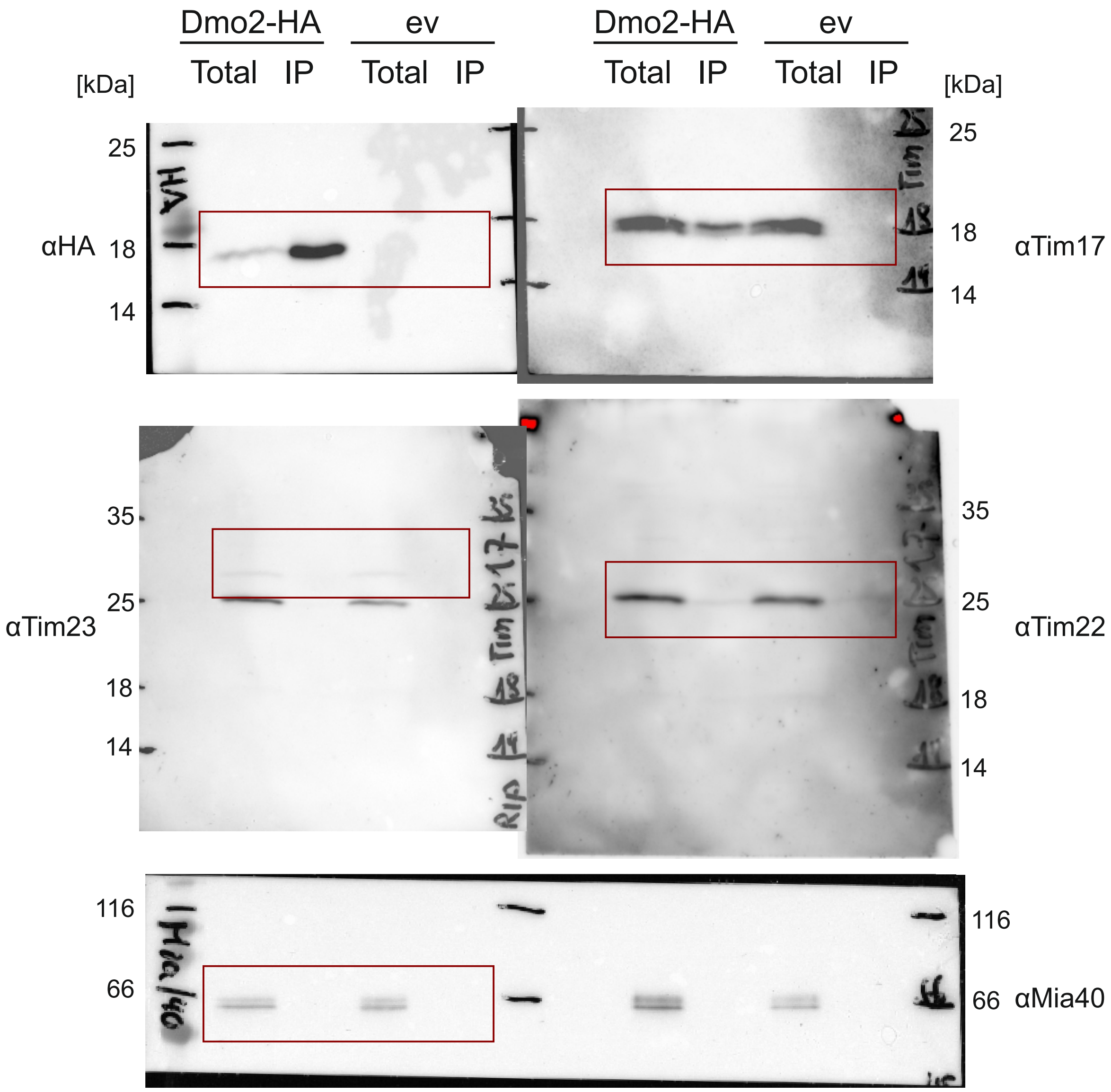
Dmo2-HA
ev
Total
IP
Total
IP
[kDa]
25
18
14
αHA
αTim17
35
25
18
14
αTim23
αTim22
116
66
αMia40

d

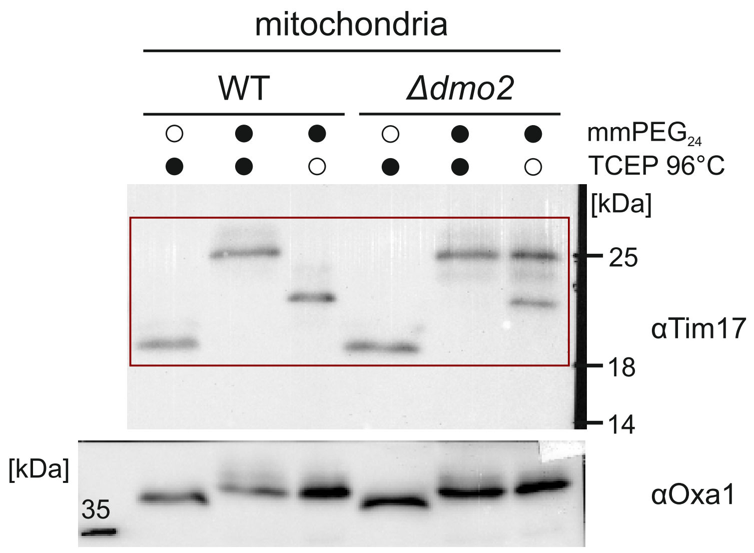
mitochondria
WT
Δdmo2
mmPEG24
TCEP 96°C
[kDa]
25
18
14
αTim17
[kDa]
35
αOxa1

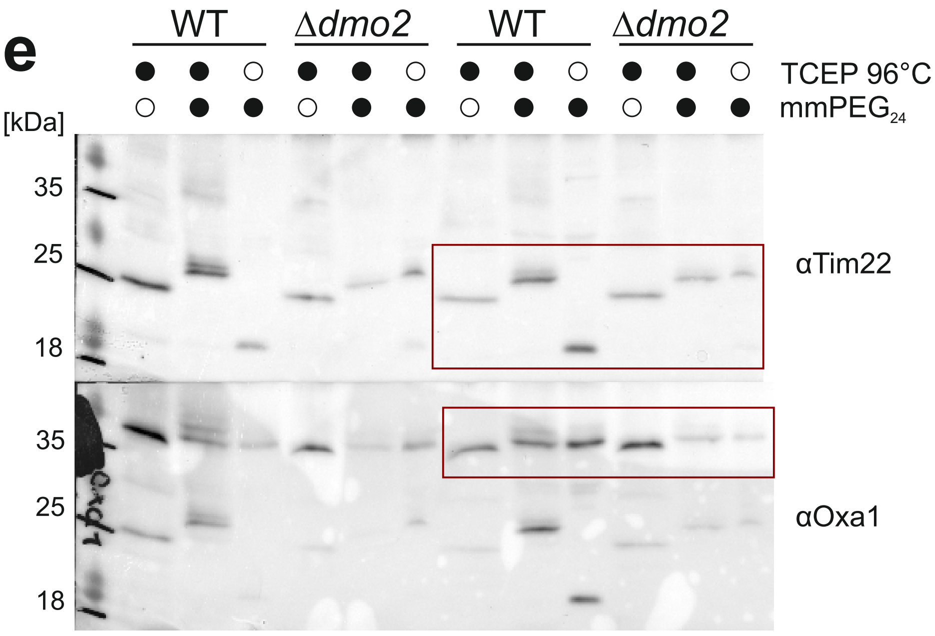
e
WT
Δdmo2
WT
Δdmo2
TCEP 96°C
mmPEG24
[kDa]
35
25
18
35
25
18
αTim22
αOxa1

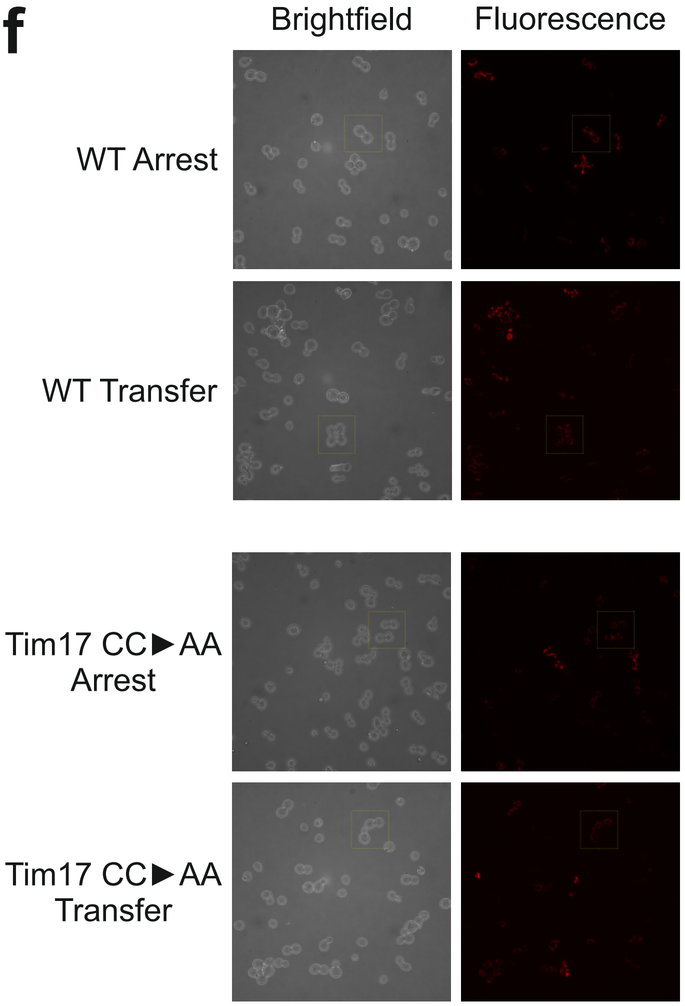
f
Brightfield
Fluorescence
WT Arrest
WT Transfer
Tim17 CC►AA Arrest
Tim17 CC►AA Transfer
