# Supplementary material for: A selectivity filter in Tim17 mediates intramitochondrial protein sorting
Source: Nat Commun. 2026 Oct 7;17:11424. doi: 10.1038/s41467-026-77990-3 (PMC13736910; doi:10.1038/s41467-026-77990-3)
Supplement: Supplementary file 5 [file 41467_2026_77990_MOESM17_ESM.zip › Source Data/Source Data Fig 6.pdf]

Figure 6

d

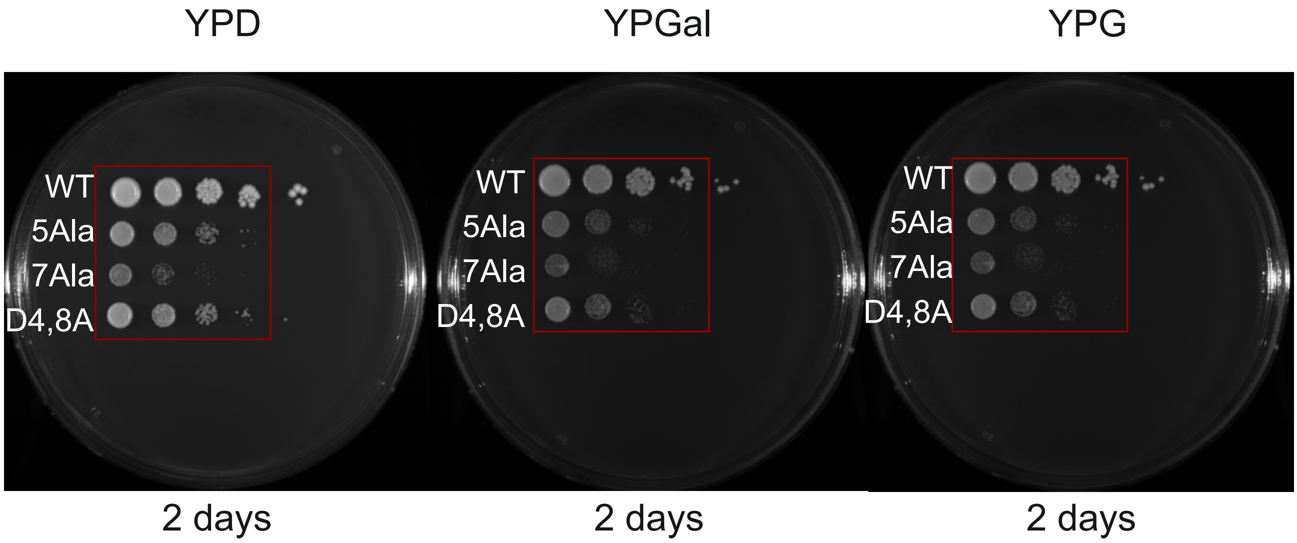

e

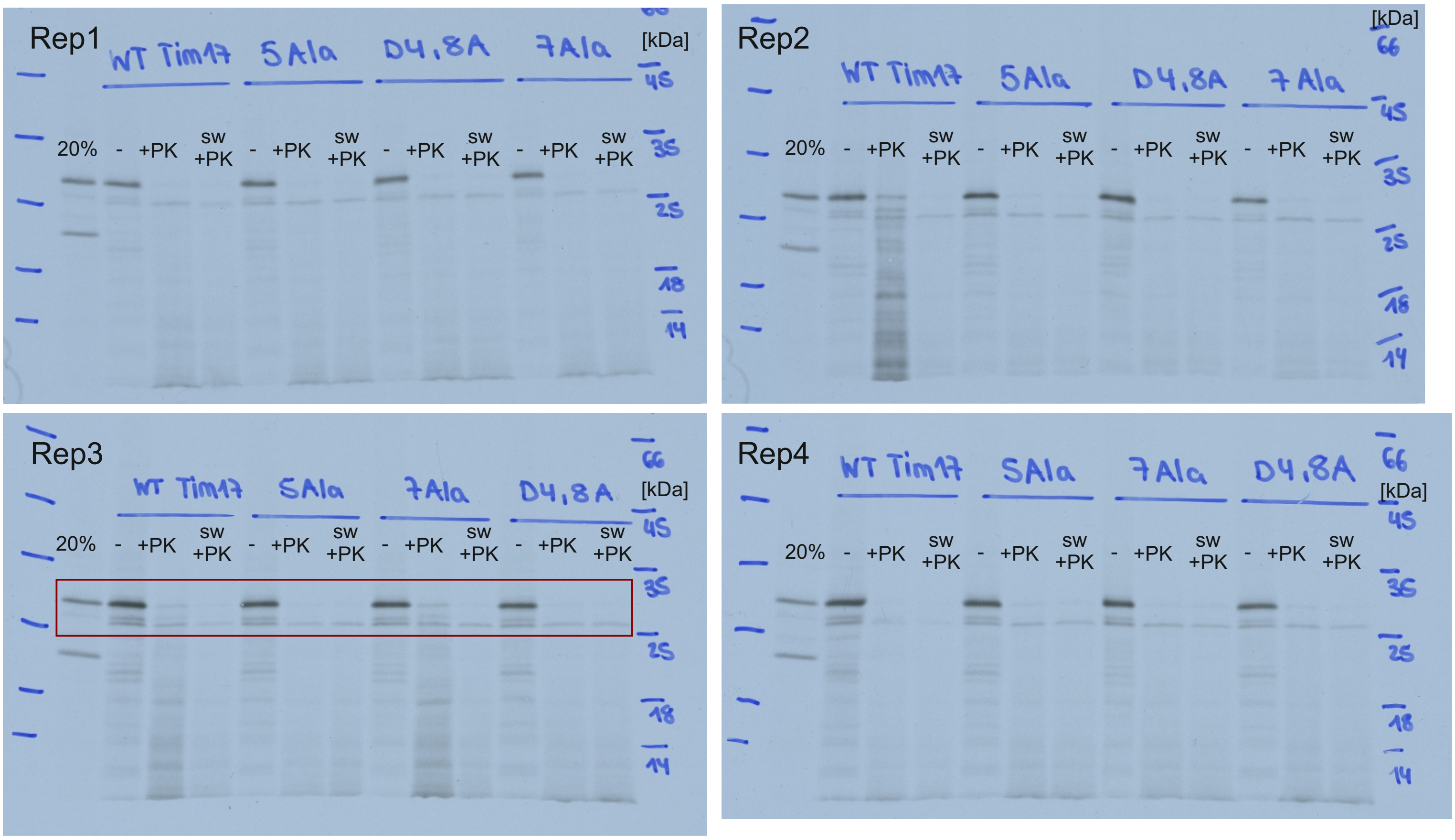

Rep 1,2,4 used for quantification

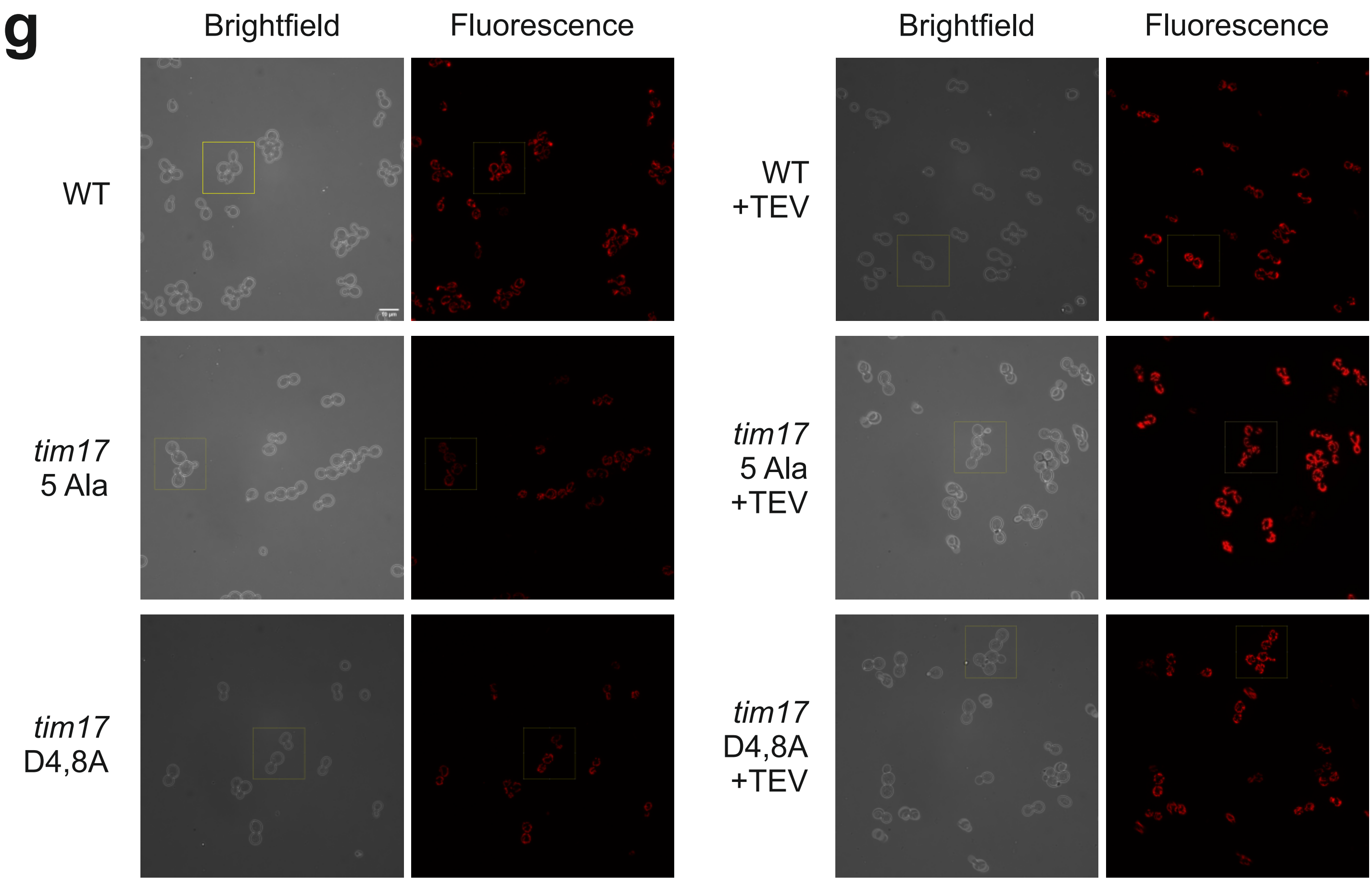

g
Brightfield
Fluorescence
Brightfield
Fluorescence
WT
WT
+TEV
tim17
5 Ala
tim17
5 Ala
+TEV
tim17
D4,8A
tim17
D4,8A
+TEV
