# Supplementary material for: A selectivity filter in Tim17 mediates intramitochondrial protein sorting
Source: Nat Commun. 2026 Oct 7;17:11424. doi: 10.1038/s41467-026-77990-3 (PMC13736910; doi:10.1038/s41467-026-77990-3)

Supplementary Figure 1

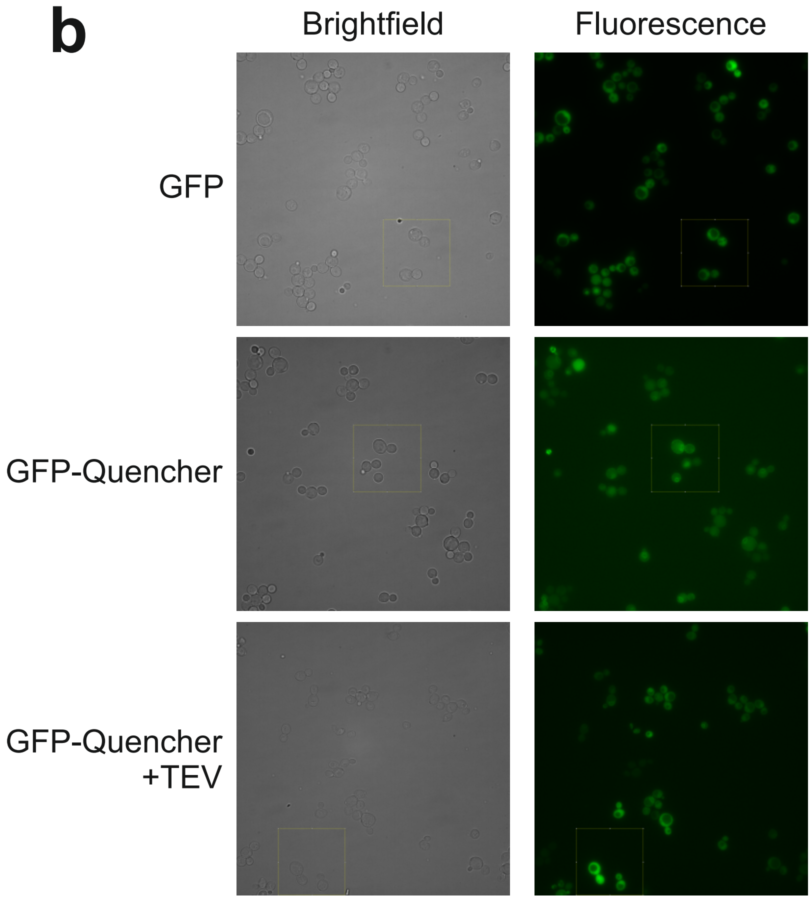

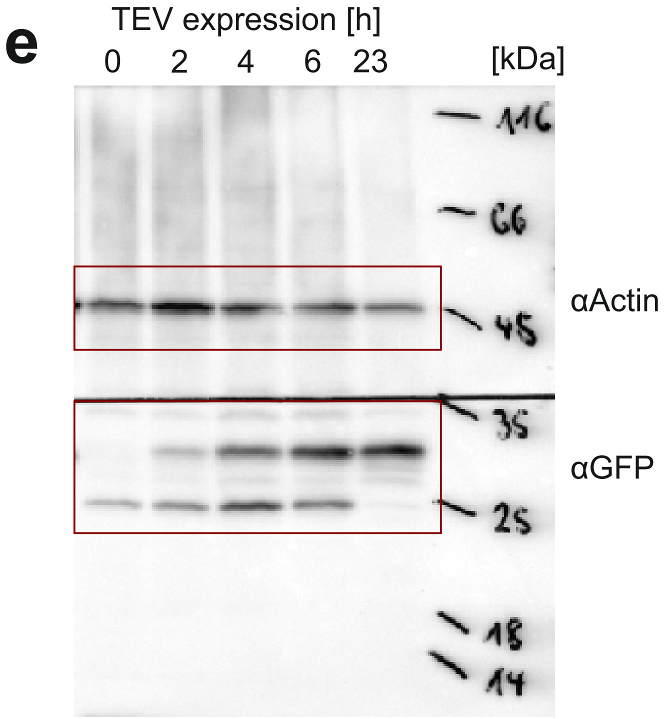

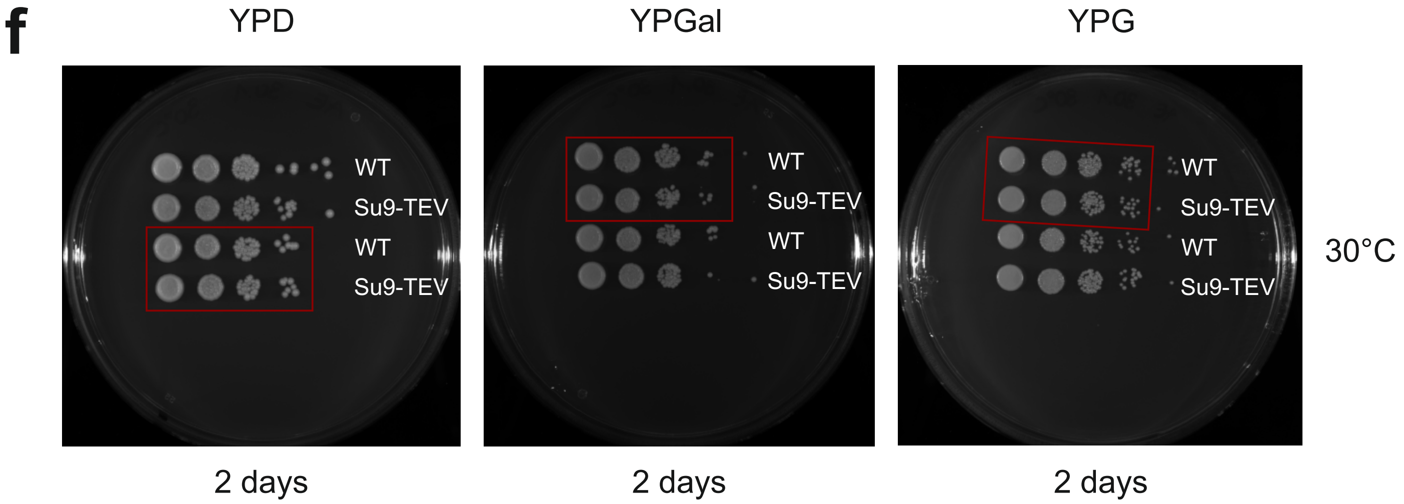

Supplement: Supplementary file 5 [file 41467_2026_77990_MOESM17_ESM.zip › Source Data/Source Data Supplementary Fig 1.pdf]
